# Evaluation of a series of nucleoside analogs as effective anticoronaviral-2 drugs against the Omicron-B.1.1.529/BA.2 subvariant: A repurposing research study

**DOI:** 10.1007/s00044-022-02970-3

**Published:** 2022-12-29

**Authors:** Amgad M. Rabie, Mohnad Abdalla

**Affiliations:** 1 Dr. Amgad Rabie’s Research Lab. for Drug Discovery (DARLD), Mansoura City 35511, Mansoura, Dakahlia Governorate Egypt; 2 Head of Drug Discovery & Clinical Research Department, Dikernis General Hospital (DGH), Magliss El-Madina Street, Dikernis City 35744, Dikernis, Dakahlia Governorate Egypt; 3 Key Laboratory of Chemical Biology (Ministry of Education), Department of Pharmaceutics, School of Pharmaceutical Sciences, Cheeloo College of Medicine, Shandong University, 44 Cultural West Road, Jinan, Shandong Province 250012 PR China

**Keywords:** Anti-SARS-CoV-2-Omicron agent, Anti-COVID-19 remedy, RNA-dependent RNA polymerase (RdRp), Proofreading 3′-to-5′ exoribonuclease, Nucleoside/Nucleotide analog, Riboprine & forodesine

## Abstract

Mysterious evolution of a new strain of the severe acute respiratory syndrome coronavirus 2 (SARS-CoV-2), the Omicron variant, led to a new challenge in the persistent coronavirus disease 2019 (COVID-19) battle. Objecting the conserved SARS-CoV-2 enzymes RNA-dependent RNA polymerase (RdRp) and 3′-to-5′ exoribonuclease (ExoN) together using one ligand is a successful new tactic to stop SARS-CoV-2 multiplication and COVID-19 progression. The current comprehensive study investigated most nucleoside analogs (NAs) libraries, searching for the most ideal drug candidates expectedly able to act through this double tactic. Gradual computational filtration afforded six different promising NAs, riboprine/forodesine/tecadenoson/nelarabine/vidarabine/maribavir. Further biological assessment proved that riboprine and forodesine are able to powerfully inhibit the replication of the new virulent strains of SARS-CoV-2 with extremely minute in vitro anti-RdRp and anti-SARS-CoV-2 EC_50_ values of about 0.21 and 0.45 μM for riboprine and about 0.23 and 0.70 μM for forodesine, respectively, surpassing both remdesivir and the new anti-COVID-19 drug molnupiravir. These biochemical findings were supported by the prior in silico data. Additionally, the ideal pharmacophoric features of riboprine and forodesine molecules render them typical dual-action inhibitors of SARS-CoV-2 replication and proofreading. These findings suggest that riboprine and forodesine could serve as prospective lead compounds against COVID-19.

**Figure:**
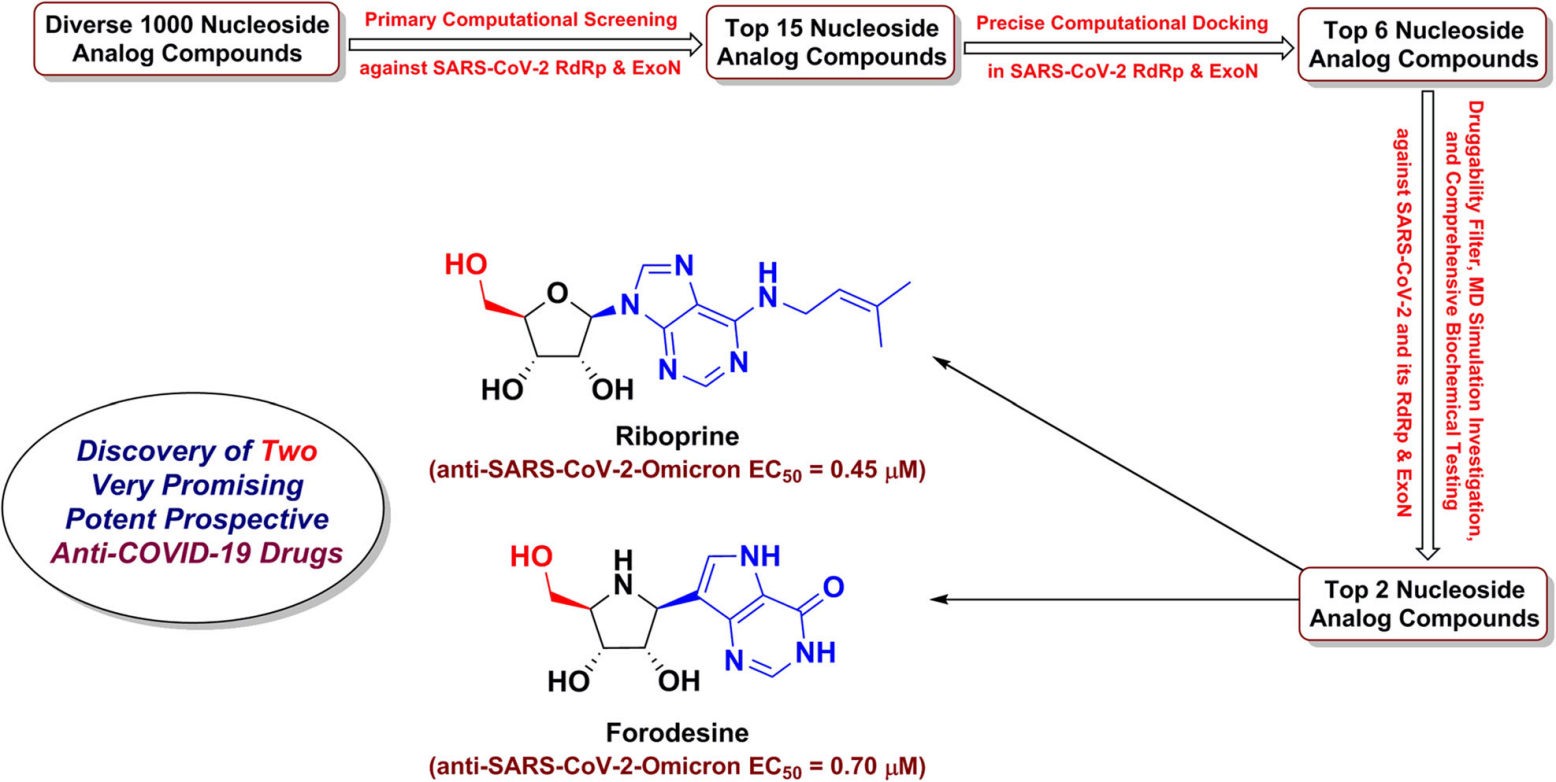
Graphical abstract

Graphical abstract

## Introduction

In the previous two years (2020–2021) since the severe acute respiratory syndrome coronavirus 2 (SARS-CoV-2) blazed across the globe, we and our multinational multidisciplinary research team have been in our laboratories day and night scrutinizing coronavirus disease 2019 (COVID-19) cases among people of different sexes/ages/races/cultures, designing new drugs against the virus, repurposing known medications against the disease, and sharing our relevant insights and visions with colleagues in Egypt, China, USA, India, and other countries. There are three principal needs that have yet to be highly met for effective and successful management of COVID-19 disease: 1) potent antiviral medications that significantly limit SARS-CoV-2 transmission, cell entry, replication, and pathogenicity, 2) medications that attenuate the acute nonproductive immune response and thus considerably decrease end-organ damage, and 3) medications that have a strong antifibrotic effect in patients with acute respiratory distress syndrome (ARDS) and thus combat the long-term sequelae of this irritating disease [1–7]. Compounds and drugs that act to satisfy mainly the first need of the three ones are relatively few to date. Of them, only nucleoside analogs (NAs) and polyphenolics (PPhs) have shown significant successful progress as SARS-CoV-2 inhibitors and killers [8–19]. By nature, NAs are more promising and highly tolerated [20]. Some new and repurposed efficacious nucleoside-like compounds are nowadays under broad investigations to be pharmacologically and clinically evaluated as effective potential anti-COVID-19 drugs, e.g., nirmatrelvir, molnupiravir, remdesivir, GS-441524, GS-443902, cordycepin, didanosine, and favipiravir, but only the first three examples reached to the clinical use stage successfully to date (only against the mild-to-moderate COVID-19 cases) [8–15].

The mysterious SARS-CoV-2 Omicron variant/lineage, also known as B.1.1.529 (or BA) variant, first began its tear around the world late 2021, and now has more than three brothers of BA subvariants, e.g., BA.1, BA.2, and BA.3 [21]. Scientists from South Africa reported the new variant on November 24, 2021, straightway after its first apparition [21]. As of 7 January of this year, 2022, the World Health Organization (WHO) reports that this highly infectious and virulent variant had been detected in more than 150 countries [21]. Omicron variant has at least 36 new mutations in its spike (S) proteins [22]. Being unfixed and changeable day by day from one strain to the newer, spike protein is not that attractive target for designing new therapies against SARS-CoV-2 variants. While, on the other hand, targeting the universal fixed proteins among all variants, e.g., SARS-CoV-2 replication RNA-dependent RNA polymerase (RdRp) and proofreading 3′-to-5′ exoribonuclease (ExoN) enzymes, through repurposing known compounds is much more effective and time-saving approach in this battle, even against the expectedly coming resistant coronaviral-2 strains. Moreover, therapies targeting the spike protein have only one chance to fight the coronaviral infection, since after passage of any viral particles inside the host body (or if these therapies were taken after the occurrence of the infection) there will not be any further abilities of these therapies to stop virus propagation and infection. Unlike therapies targeting the replication and proofreading enzymes, which have unlimited number of continuous chances to fight the virus and its successors, and prevent their further multiplication throughout the entire human body (even if these therapies were taken after the occurrence of the infection). In the first weeks of 2022, we as a multidisciplinary team continued our scientific journey and worked around the clock to discover effective anti-SARS-CoV-2-Omicron-variant drug candidates.

**Fig. 1:**
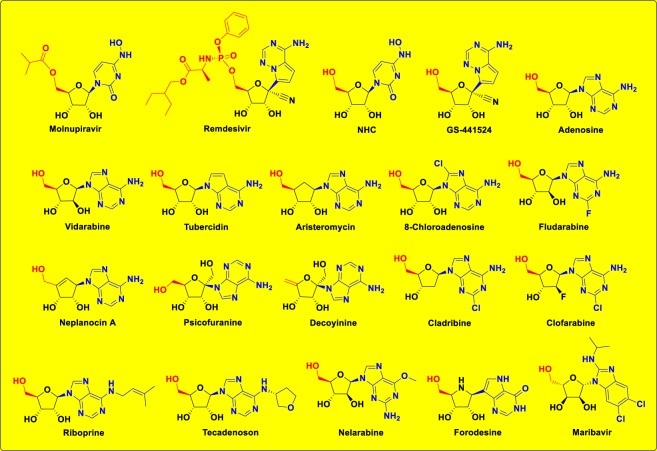
Molecular structures of the four reference anti-SARS-CoV-2 drugs (molnupiravir, remdesivir, NHC, and GS-441524) along with adenosine, shown in the first row, and molecular structures of the fifteen examined NAs (in the newly-designed small chemical library) as prospective anti-SARS-CoV-2 drugs, shown in the second to fourth rows

Tactical nucleoside analogism is among the favorable therapeutic choices in drug designers’ and pharmaceutical chemists’ brains to fight and stop the coronavirus multiplication inside the human body [9–15, 20]. In this COVID-19 therapeutic tactic the used nucleoside/nucleotide analog makes use of its close similarity with the normal natural nucleosides and nucleotides to misguide and deceive the SARS-CoV-2 RdRp (the nonstructural protein complex 12/7/8 or nsp12-nsp7-nsp8) and ExoN (the nonstructural protein complex 14/10 or nsp14-nsp10) enzymes [20]. Nsp12-nsp7-nsp8 and nsp14-nsp10 protein complexes are very indispensable enzymes in the replication/proofreading of the coronaviral-2 genome, and thus, their strong inhibition will significantly block the replication of SARS-CoV-2 particles. Nucleoside-like agents confuse both RdRp and ExoN enzymes through complete incorporation in the viral RNA genetic strands in place of the correct naturally-occurring nucleosides/nucleotides, resulting in repeated excessive ambiguous coding and premature termination of RNA synthesis with the formation of vague RNA strands at the end; these faulty strands represent abnormal noninfectious and inactive particles, hence there would not be any further multiplication of the virus [13, 14, 20]. Some of the aforementioned anti-COVID-19 agents, e.g., molnupiravir, remdesivir, and their active metabolites, β-D-*N*^4^-hydroxycytidine (NHC or EIDD-1931) and GS-441524, respectively (Fig. 1), count on this effective mechanism in their inhibitory and blocking activities on the SARS-CoV-2 particles [9–12]. With the progressive evolution of more resistant new strains/variants of SARS-CoV-2, discovering more potent and broad-spectrum natural or synthetic anti-SARS-CoV-2 drugs became a must.

In this current research work, we have explored the combined inhibitory activities of some NAs on both SARS-CoV-2 RdRp and ExoN enzymes as a novel effective strategy to double combat COVID-19 [23]. After screening of different libraries of nucleosides and NAs, we chose the top fifteen nucleoside-like compounds with the best results to make a very small library of them specifically designed for our work (Fig. 1). Almost all the selected top NAs were adenosine analogs (adenosine structure is shown in Fig. 1). Computation-based molecular docking revealed that about six of these fifteen compounds showed very good binding free energies with both enzymes, SARS-CoV-2 RdRp and ExoN, compared to those of the two positive controls (references), remdesivir and molnupiravir, with the same two enzymes. However, the other compounds of the fifteen ones, e.g., neplanocin A, tubercidin, and fludarabine, showed relatively moderate-to-good results. Molecular docking and dynamics simulations studies of the chosen six compounds disclosed the superiority of the two compounds riboprine and forodesine in hitting the catalytic active sites of both enzymes with the formation of much more stable complexes having higher negative binding free energies. Biological evaluations of the six NAs against both coronaviral-2 RdRp and ExoN proteins and against the entire SARS-CoV-2 Omicron variant particles demonstrated nearly the same interesting superiority of riboprine and forodesine, respectively.

Based on these current results and previous data [24–27], we can conclude that, first, riboprine and forodesine can be further in vivo and clinically investigated for repurposing against COVID-19 and, second, the expected potent clinical inhibitory effects of riboprine and forodesine against SARS-CoV-2 replication may be principally attributed to the triple synergistic inhibitory activities against the three enzymes RdRp, ExoN, and adenosine kinase (ADK), i.e., may be closely related to RdRp, ExoN, and ADK inhibitory activities of riboprine and forodesine. The possible SARS-CoV-2 RNA mutagenicity of both drugs *via* nucleoside analogism mode of action and incorporation into the new coronaviral-2 RNA strands should also be extensively and clinically studied. The pharmacokinetics of these drugs which we intend to try repurposing them against COVID-19 should be significantly put into account, because tissue distributions of these potential anticoronaviral-2 drugs will certainly affect their total capabilities of reducing viral loads of SARS-CoV-2 particles in COVID-19 therapy [28]. The possibility of pharmaceutically formulating the promising nucleoside-like agents of the six tested ones as rapid-action nasal/oral anti-COVID-19 spray/drops should also be considered.

## Results and discussion

### Computational molecular modeling of the selected NAs as prospective anti-COVID-19 drugs

After computational screening and filtration of several libraries of nucleosides and NAs, the top fifteen nucleoside-like molecules with the best and most ideal pharmacodynamic/pharmacokinetic results with respect to the predicted anti-SARS-CoV-2 activities were chosen for our specific mission. The selected compounds (the finalists) were, respectively, as follows: riboprine, forodesine, tecadenoson, nelarabine, vidarabine, maribavir, neplanocin A, tubercidin, cladribine, decoyinine, aristeromycin, fludarabine, clofarabine, psicofuranine, and 8-chloroadenosine. A small new library was made of these fifteen compounds which are a mixture of natural and synthetic molecules (Fig. 1). In a next step, further molecular docking specifically against SARS-CoV-2 RdRp and ExoN revealed that the compounds riboprine, forodesine, tecadenoson, nelarabine, vidarabine, and maribavir, respectively, have the lowest and best inhibitory binding energies (ranged from −6.4 to −7.8 kcal/mol) compared to the two reference anti-RdRp/anti-ExoN drugs, remdesivir and molnupiravir (having binding energies ranged from −6.3 to −7.3 kcal/mol), as shown in Table 1. The catalytic pockets (i.e., active sites) of the two coronaviral-2 enzymes, RdRp (which is the main enzyme responsible for replication and transcription of the coronaviral-2 RNA genome) and ExoN (it is worth mentioning that nsp14 or the proofreading exoribonuclease of SARS-CoV-2 has two active sites; the exoribonuclease active site, the major one that we are concerned with in the current study, and the methyltransferase active site), were nearly detected and validated through previous several computational, crystallographic, and biochemical experiments in the literature [29–32]. Investigating and analyzing the resultant in silico interactions of the aforementioned six molecules with the residues of SARS-CoV-2 RdRp and ExoN proteins showed that all molecules significantly hit most of the active amino acid residues of the catalytic pockets of both enzymes with strong interactions, including, mainly, hydrogen bonding (H-bonds), hydrophobic interactions, ionic bonds, and water bridges (weaker in some examples), of relatively short bond distances and low binding energies.

**Table 1:** The binding affinity energy values (docking S-scores) estimated during molecular docking of the fifteen screened NAs against the two SARS-CoV-2 proteins, RdRp and ExoN enzymes (using remdesivir and molnupiravir as the positive control drugs). The fifteen NAs are arranged in a collective descending order, beginning from the top ranked one and ending with the least ranked one

Classification	Compound Name	Docking S-score (kcal/mol)	
		RdRp (7BV2)	ExoN (7MC6)
*Screened NAs*	Riboprine	−7.2	−7.8
	Forodesine	−7.4	−7.6
	Tecadenoson	−7.1	−7.5
	Nelarabine	−7.5	−6.9
	Vidarabine	−7.2	−6.7
	Maribavir	−6.4	−7.4
	Neplanocin A	−7.1	−6.5
	Tubercidin	−6.9	−6.6
	Cladribine	−6.9	−6.6
	Decoyinine	−6.2	−7.1
	Aristeromycin	−6.0	−7.1
	Fludarabine	−6.2	−6.8
	Clofarabine	−6.1	−6.8
	Psicofuranine	−6.1	−6.7
	8-Chloroadenosine	−5.9	−6.9
*Reference Drugs*	Remdesivir	−6.5	−7.0
	Molnupiravir	−6.3	−7.3

**Fig. 2:**
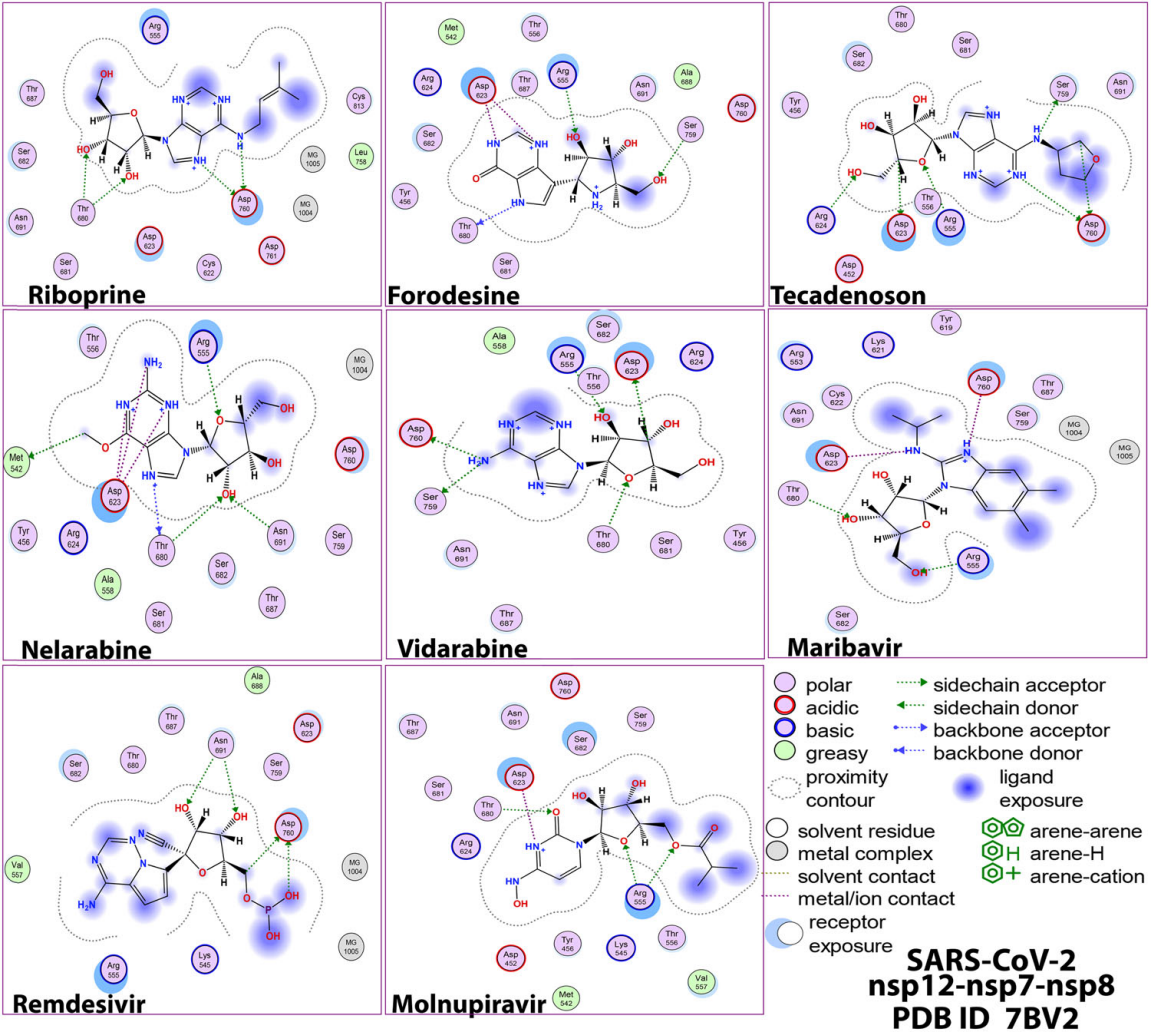
2-D images of the postdocking interactions of the six NAs, riboprine, forodesine, tecadenoson, nelarabine, vidarabine, and maribavir, and the two reference drugs, remdesivir and molnupiravir, respectively, with the SARS-CoV-2 RdRp “nsp12” enzyme cocrystallized with its protein cofactors nsp7 and nsp8 (PDB ID: 7BV2)

**Fig. 3:**
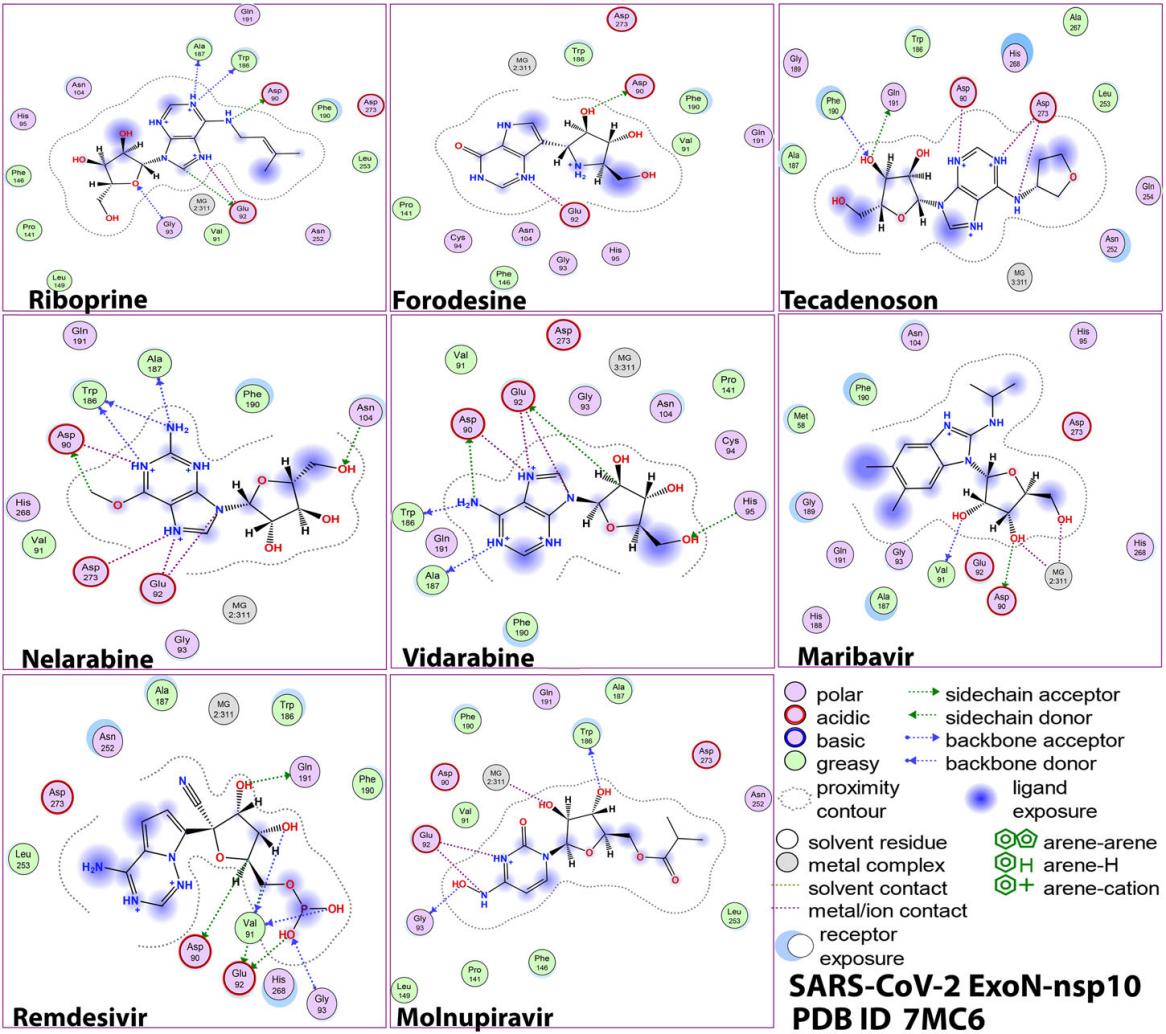
2-D images of the postdocking interactions of the six NAs, riboprine, forodesine, tecadenoson, nelarabine, vidarabine, and maribavir, and the two reference drugs, remdesivir and molnupiravir, respectively, with the SARS-CoV-2 ExoN “nsp14” enzyme cocrystallized with its protein cofactor nsp10 (PDB ID: 7MC6)

**Fig. 4:**
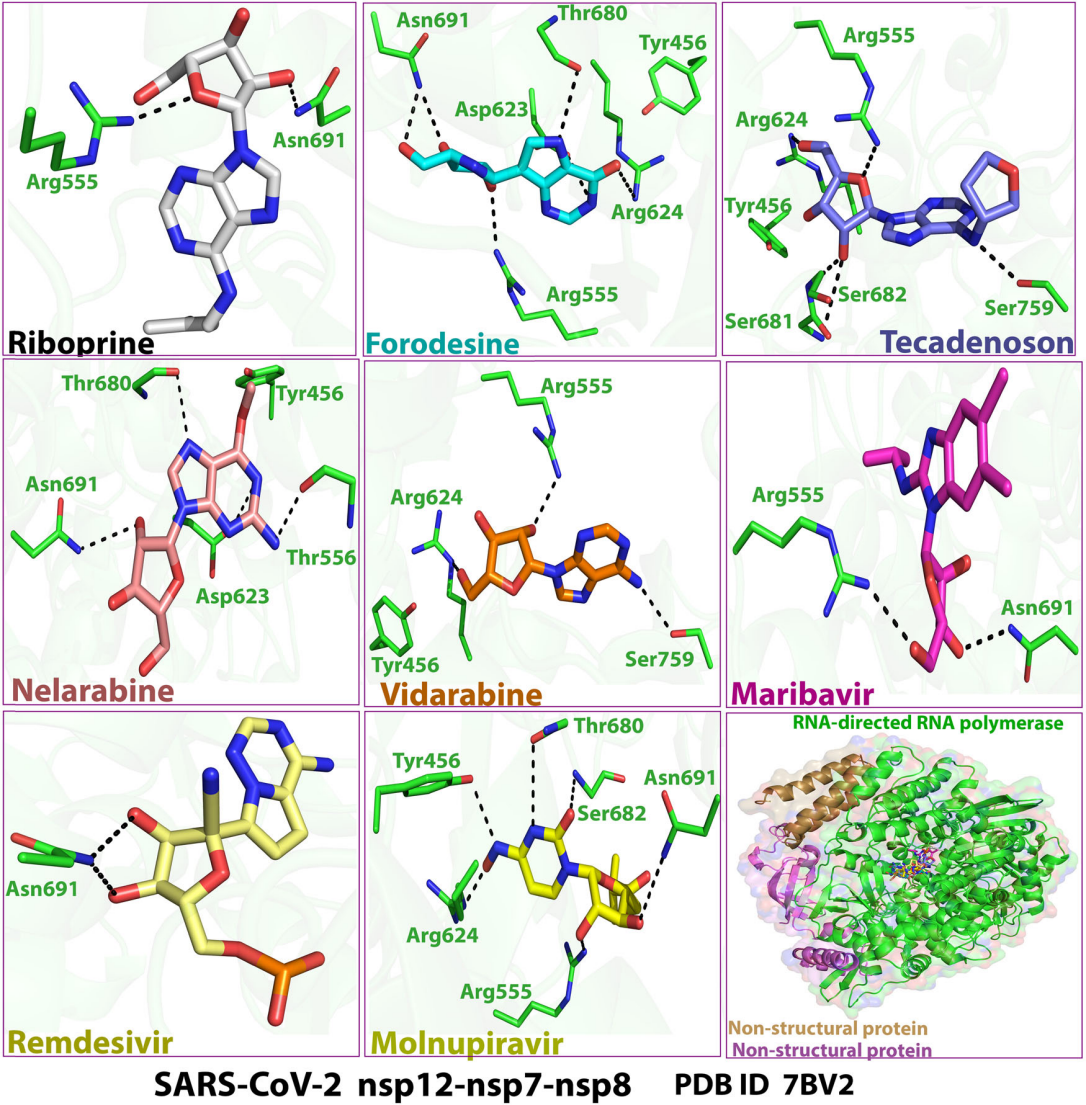
3-D images of the postdocking interactions of the six NAs, riboprine, forodesine, tecadenoson, nelarabine, vidarabine, and maribavir, and the two reference drugs, remdesivir and molnupiravir, respectively, with the SARS-CoV-2 RdRp “nsp12” enzyme cocrystallized with its protein cofactors nsp7 and nsp8 (PDB ID: 7BV2)

**Fig. 5:**
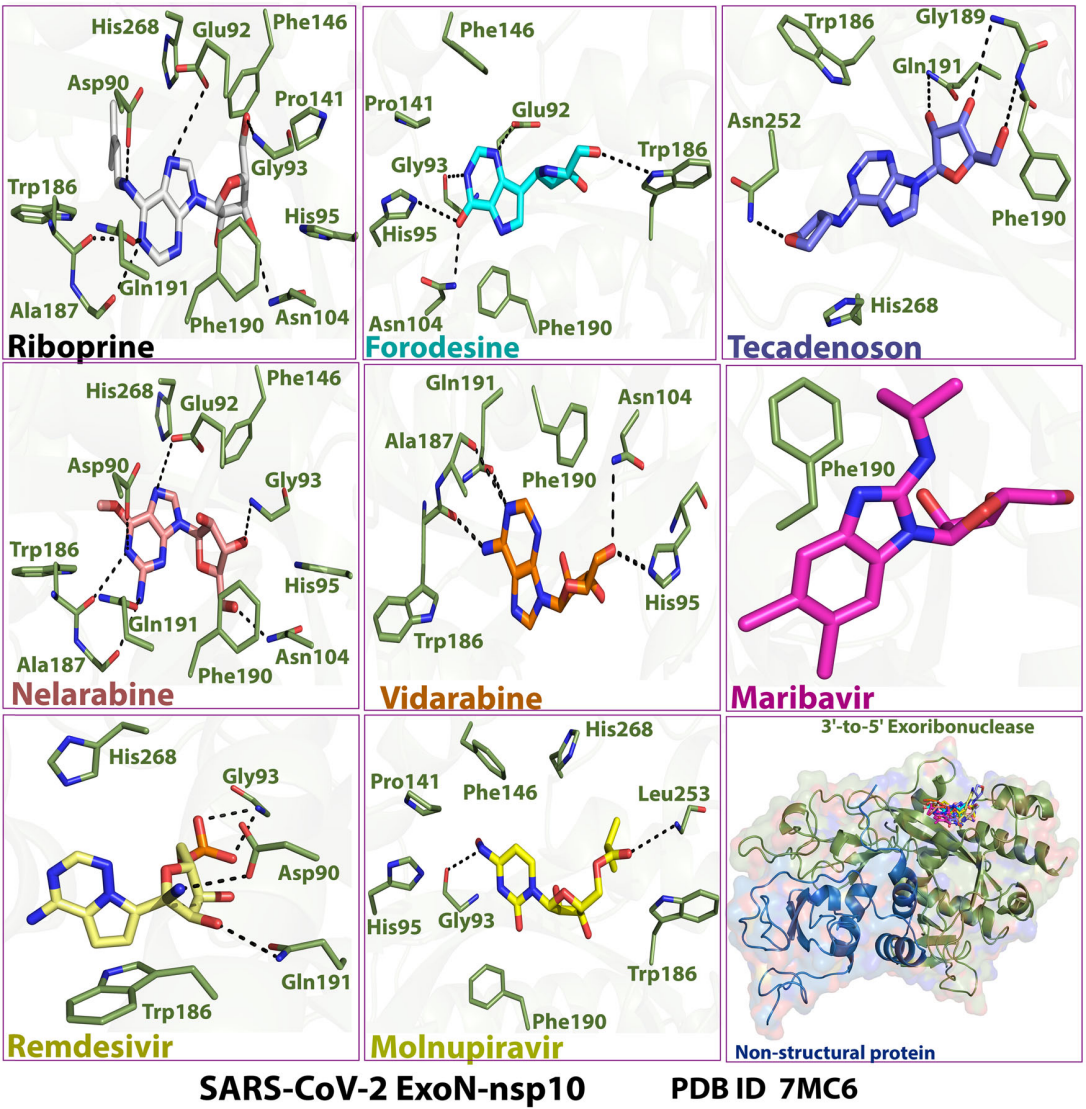
3-D images of the postdocking interactions of the six NAs, riboprine, forodesine, tecadenoson, nelarabine, vidarabine, and maribavir, and the two reference drugs, remdesivir and molnupiravir, respectively, with the SARS-CoV-2 ExoN “nsp14” enzyme cocrystallized with its protein cofactor nsp10 (PDB ID: 7MC6)

The extracted images in Figs. 2–5 show the detailed two-dimensional (2-D) and three-dimensional (3-D) representations of the most evident intermolecular interactions between each ligand of the six ones and each of the two coronaviral-2 enzymes, respectively. The 3-D representations focus mostly on the shortest bonds. The molecules of these six NAs strongly strike most of the neighboring active residues of the major catalytic pocket of SARS-CoV-2 RdRp (in chain A, i.e., 7BV2-A receptor), e.g., Arg553, Arg555, Thr556, Ala558, Lys621, Cys622, Asp623, Arg624, Thr680, Ser681, Ser682, Thr687, Ala688, Asn691, Leu758, Ser759, Asp760, Asp761, and Cys813. On the other hand, the molecules of the same six NAs powerfully strike most of the adjacent active residues of the major catalytic pocket (exoribonuclease active site) of SARS-CoV-2 ExoN (in chain A; QHD43415_13 receptor), e.g., Asp90, Val91, Glu92, Gly93, Cys94, His95, Asn104, Pro141, Phe146, Leu149, Trp186, Ala187, Gly189, Phe190, Gln191, Asn252, Leu253, Gln254, His268, and Asp273. These interactions are very promising and very comparable to, or even in some cases significantly better than, those of remdesivir and molnupiravir with the same two enzymes.

Analysis of the molecular dynamics (MD) simulation results revealed the relative stabilities of the formed protein-ligand complexes of each of the six NAs with each of the two enzymes when compared with the reference drugs. Complexes of the NAs with SARS-CoV-2 ExoN are slightly more stable, with less numbers/intensities of fluctuations, and with lower root-mean-square deviation (RMSD) and root-mean-square fluctuation (RMSF) values than those with SARS-CoV-2 RdRp. Interestingly, riboprine and forodesine displayed the best results among all in most of the compared MD items during the simulation. Comprehensively, the RdRp-riboprine, RdRp-forodesine, ExoN-riboprine, and ExoN-forodesine complexes appear to be reasonably stable. The early fluctuations (which were not mostly extreme) in RMSF and RMSD trajectories may be indications of some conformational changes within the enzyme complex system as a result of the adequate repositioning of both ligands inside the catalytic binding sites which takes some nanotime till the formation of very interesting strong molecular interactions. Possible unrevealed allosteric modulations, specially in case of the larger protein complex SARS-CoV-2 nsp12-nsp7-nsp8, could also be put into consideration. Forodesine has the lowest radius of gyration (rGyr) values (less than 3.5 Å) among all the tested compounds, including the references, with both enzymes, indicating more compact and stable protein systems. In addition, from the computational point of view, forodesine followed by riboprine have the best balanced molecular surface area (MolSA), solvent-accessible surface area (SASA), and polar surface area (PSA) values among all the investigated compounds. Interestingly, riboprine displayed the largest interactions fraction (of more than 2% of the total interactions predicted) of the strong H-bonds with the docked SARS-CoV-2 proteins, among all the tested compounds, and this occurs specifically with the catalytic amino acid residue Asp90 in the small protein SARS-CoV-2 nsp14-nsp10 in its relatively stable complex with riboprine, indicating a significant potential of riboprine to give a strongly-inhibited/blocked status of the ExoN enzyme. MD simulation results also confirmed nearly all the primary molecular docking data with regard to, for example, the interacting amino acids along with the numbers/types/strengths of the formed bonds. In the Supplementary Material file, Figs. S1–S10 show the detailed results of MD simulation of the interactions between each ligand of the most promising NAs, riboprine and forodesine, with each of the two coronaviral-2 enzymes, RdRp and ExoN, respectively (in comparison with the two reference FDA-approved anti-SARS-CoV-2 RdRp drugs, remdesivir and molnupiravir). The previous computational data were very encouraging to motivate us to transfer to the biological evaluation part of the current work.

### Experimental biological evaluation of the selected NAs as prospective anti-COVID-19 drugs

The first preclinical assay in this extensive assessment is the robust cell-based test, the in vitro anti-SARS-CoV-2 RdRp bioassay, which was recently developed using Gaussia-luciferase (Gluc) as the reporter to assess the anticoronaviral-2 RdRp activity of mainly the NAs (the prodrugs of nucleotides) without any necessity for generating the active nucleotidic triphosphate forms of the NAs (or of the other nontriphosphorylated nucleotidic analogs, i.e., of the monophosphorylated and diphosphorylated NAs) as for the cell-free assays [24, 25]. Moreover, it was undoubtedly confirmed, through the findings of this new biochemical assay, that the exonuclease activity of SARS-CoV-2 nsp14 significantly improves the SARS-CoV-2 RdRp resistance to the various inhibitors of the nucleoside/nucleotide analogs class (one of the primary factors that aggravates the resistance and severe pathogenicity of SARS-CoV-2 particles are their abilities to encode the nsp14 ExoN which is capable of taking off the faulty mutagenic nucleotides misincorporated by the low-fidelity RdRp into the growing coronaviral-2 RNA strands, causing considerable resistance to nucleos(t)ide analog therapeutic agents), thus ExoN effects were considered and added in the steps of this screening assay protocol which was primarily designed for exploring possible SARS-CoV-2 RdRp inhibitors (dissimilar to the traditional analytical cell-free assay) [24, 25, 33, 34]. The assay can be metaphorically called “anti-SARS-CoV-2 RdRp/ExoN bioassay”.

As previously mentioned, we mainly concentrate here on the two principal protein complexes that catalyze and control the SARS-CoV-2 replication/transcription processes, nsp12-nsp7-nsp8 polymerase complex and nsp14-nsp10 exoribonuclease complex, respectively. This test significantly simulates the respective original replication processes that occur for the SARS-CoV-2 genome, as it functionally mimics the RNA generating processes driven mainly by the SARS-CoV-2 RdRp [35]. Table 2 displays the detailed values obtained from this in vitro anti-SARS-CoV-2 RdRp/ExoN bioassay. The resultant data showed that, among the tested target NAs, riboprine and forodesine demonstrated the best results. The two compounds effectively inhibited SARS-CoV-2 RdRp activity with very excellent small EC_50_ values of 0.21 and 0.23 μM, which very slightly increased in the presence of SARS-CoV-2 ExoN (the wild type) to about 0.30 and 0.33 μM, respectively, indicating the potent inhibitory/blocking activities of both compounds against SARS-CoV-2 ExoN, which appeared in these extremely minute nanomolar differences of the EC_50_ values between both cases. Mutations in the exoribonuclease (i.e., the mutated type; e.g., D90A/E92A mutations of the active catalytic residues in nsp14 as in our current case) reinforced the anti-RdRp activity of riboprine and forodesine to excellent EC_50_ values of 0.25 and 0.27 μM (i.e., slightly lower than that resulted in the presence of the normal wild type of ExoN; these very slight changes also reflected, as previously mentioned, the potent activities of both NAs against SARS-CoV-2 ExoN in its original wild type from the beginning prior to any intended mutations). These previous values of riboprine and forodesine even surpassed those of the two potent reference agents, remdesivir and molnupiravir, which showed higher values, reflecting the possible superiority of both NAs over remdesivir/molnupiravir in clinical investigation in humans. The results also proved that molnupiravir and remdesivir could not resist the performance of Omicron variant ExoN the same way and potency riboprine and forodesine do. The other target NAs, nelarabine, tecadenoson, maribavir, and vidarabine, also showed very good promising and small values, but with less degree than those of riboprine, forodesine, and the reference molnupiravir, respectively. It is apparently observed from the values in Table 2 that as much the EC_50_ values of the NA against the polymerase alone and against the polymerase in the presence of the exoribonuclease are close to each other, as more potent this NA inhibitor is (i.e., as more predicted for this tested NA to be an ideally effective RdRp inhibitor or, more accurately, SARS-CoV-2 replication inhibitor). From the results we can also conclude that an ideal potent SARS-CoV-2 RdRp inhibitor should have a ratio of EC_50*(polymerase + exoribonuclease)*_/EC_50*(polymerase)*_ that is very close to 1 and less than 2. The lower this ratio, the more likely the tested compound to ideally inhibit SARS-CoV-2 replication. Riboprine displayed the highest resistance, among all the tested compounds, to the coronaviral-2 nsp14 exoribonuclease activity in HEK293T cells. The very promising capabilities of riboprine and forodesine to inhibit the nsp12 polymerase and nsp14 exoribonuclease activities of the coronaviral-2 Omicron variant interestingly uphold the repurposing potentials of riboprine and forodesine in clinical settings for further therapeutic use as potent anti-COVID-19 drugs. It is worth mentioning that riboprine and forodesine are nearly the only NAs that have such unique potent anti-SARS-CoV-2 activities against both the RdRp and ExoN enzymes of the newest SARS-CoV-2 variant, the Omicron variant, in very significant values to date (this is to the best of our current knowledge during the submission of this research paper for publication) [24, 25]. These present biochemical findings concerning the potent inhibitory SARS-CoV-2 RdRp-binding and ExoN-binding properties of riboprine and forodesine are in an ideal agreement with almost all the computed parameters of the prior in silico part of this comprehensive research, which was discussed in details in Computational molecular modeling of the selected NAs as prospective anti-COVID-19 drugs.

**Table 2:** Anti-SARS-CoV-2 RdRp/ExoN activities (along with respective ratios) of the target repurposed drugs riboprine, forodesine, nelarabine, tecadenoson, maribavir, and vidarabine (using both remdesivir and molnupiravir as the positive control/reference drugs, and dimethylsulfoxide “DMSO” as the negative control/placebo drug), respectively, in HEK293T cells, expressed as EC_50_ values in μM (please note that, in this table, nsp12 refers to nsp12/7/8 complex, nsp14 refers to nsp14/10 complex, and nsp14_mutant_ refers to nsp14_mutant_/10 complex)

Classification	Compound Name	Inhibition of SARS-CoV-2 RdRp in vitro (EC_50_ in μM)^a^			Respective Ratios of EC_50_	
		Nsp12	Nsp12 + Nsp14	Nsp12 + Nsp14_mutant_	(Nsp12 + Nsp14)/Nsp12	(Nsp12 + Nsp14_mutant_)/Nsp12
*Repurposed NAs*	Riboprine	0.21 ± 0.03	0.30 ± 0.03	0.25 ± 0.03	1.43	1.19
	Forodesine	0.23 ± 0.03	0.33 ± 0.04	0.27 ± 0.03	1.44	1.17
	Nelarabine	0.63 ± 0.05	1.20 ± 0.07	1.07 ± 0.06	1.91	1.70
	Tecadenoson	0.95 ± 0.06	1.34 ± 0.08	1.26 ± 0.07	1.41	1.33
	Maribavir	1.05 ± 0.06	1.88 ± 0.08	1.43 ± 0.08	1.79	1.36
	Vidarabine	1.07 ± 0.05	2.00 ± 0.07	1.44 ± 0.05	1.87	1.35
*Reference Drugs*	Remdesivir	1.12 ± 0.06	2.10 ± 0.08	1.56 ± 0.07	1.88	1.39
	Molnupiravir	0.28 ± 0.03	0.48 ± 0.04	0.35 ± 0.04	1.71	1.25
*Placebo Solvent*	DMSO	>100	>100	>100	N.A.^b^	N.A.

^a^EC_50_ or 50% effective concentration is the concentration of the tested compound that is required for 50% reduction in the COVID-19 polymerase (SARS-CoV-2 RdRp) activity in vitro. EC_50_ is expressed in μM

^b^N.A. means not available (i.e., it was not determined)

The second assay is the collective in vitro anti-SARS-CoV-2 and cytotoxicity tests. Table 3 shows the resultant values from both tests in details. The used SARS-CoV-2 strain in the anticoronaviral-2 assay is the new variant of SARS-CoV-2, the Omicron variant B.1.1.529/BA.2 sublineage, which is one of the most infectious and resistant strains of the virus. The data displayed in the table interestingly revealed the significantly higher antiviral efficacies of each of the two NAs riboprine and forodesine against the newly-appeared variants of SARS-CoV-2 as compared to those of each of the two positive control reference drugs remdesivir and molnupiravir (the placebo drug DMSO showed extremely weak activities, i.e., negligible results). Riboprine and forodesine were found to efficiently inhibit and impair the entire SARS-CoV-2 replication/transcription in Vero E6 cells with EC_50_ values extremely smaller than the 100 μM value of stock concentration, continuing their superiorities over the other tested target NAs exactly as in the previous anti-RdRp/ExoN biochemical assay. Promisingly, the natural NA riboprine was proved to be very leading (i.e., ranked first among all the tested compounds) in its total anti-Omicron activity (EC_50_ = 0.45 μM), which was found to be 4.6 and 5.8 times as effective as the two reference drugs, remdesivir (EC_50_ = 2.07 μM) and molnupiravir (EC_50_ = 2.61 μM), respectively, with respect to the tested in vitro anti-B.1.1.529-BA.2/anti-SARS-CoV-2 activity. While forodesine was ranked second, among all the tested compounds, in its total anti-Omicron activity (EC_50_ = 0.70 μM), which was found to be about 2.96 and 3.73 times as effective as the two reference drugs, remdesivir and molnupiravir, respectively, with respect to the same evaluated activity. According to the current cytotoxicity assay, the in vitro CC_50_ values of riboprine and forodesine are significantly greater than 100 μM, therefore these two NAs are expected to have very advantageous high corresponding clinical selectivity indices “SIs” (SI_riboprine_ > 222.2 and SI_forodesine_ > 142.9; while remdesivir and molnupiravir have narrower SIs, SI_remdesivir_ > 48.3 and SI_molnupiravir_ > 38.3), reflecting the specific/selective anti-RNA actions of the riboprine and forodesine molecules against the new coronaviral-2 Omicron genome rather than the human genome. Riboprine and forodesine displayed significantly small values of the concentration that results in 100% in vitro inhibition of the coronaviral-2 Omicron variant cytopathic effects (CPEIC_100_ = 1.21 and 1.69 μM, respectively), which are less than the corresponding values of remdesivir (CPEIC_100_ = 5.99 μM) and molnupiravir (CPEIC_100_ = 6.28 μM) and also less than those of the other tested NAs. In line with their potent activities against the infectious coronaviral-2 B.1.1.529/BA.2 substrain, riboprine and forodesine also showed very slight values of the concentration that is needed for 50% in vitro lowering in the number of RNA copies of the B.1.1.529/BA.2 substrain of SARS-CoV-2 (0.48 and 0.73 μM, respectively), which are clearly smaller than the corresponding values of both remdesivir and molnupiravir (2.11 and 2.73 μM, respectively). EC_90_ values for riboprine and forodesine, which are preferably used for the in vivo/clinical studies, were also very small and consistent with the EC_50_ values (being not far that much from the EC_50_ values indicates the expected significant clinical potencies of both drugs) as demonstrated in Table 3. Nelarabine, tecadenoson, maribavir, and vidarabine displayed slightly higher concentration values (EC_50_, EC_90_, CC_50_, and CPEIC_100_) than those displayed by riboprine and forodesine, but still comparable to those of the positive control drugs, remdesivir and molnupiravir.

**Table 3:** Anti-SARS-CoV-2/anti-COVID-19 activities (along with cytotoxicities) of the target repurposed drugs riboprine, forodesine, nelarabine, tecadenoson, maribavir, and vidarabine (using both remdesivir and molnupiravir as the positive control/reference drugs, and DMSO as the negative control/placebo drug), respectively, against SARS-CoV-2 (Omicron variant, B.1.1.529/BA.2 sublineage) in Vero E6 cells

Classification	Compound Name	CC_50_^a^ (μM)	Inhibition of SARS-CoV-2 Replication in vitro (Anti-B.1.1.529/BA.2 Bioactivities) (μM)			
			100% CPE Inhibitory Concentration (CPEIC_100_)^b^	50% Reduction in Infectious Virus (EC_50_)^c^	50% Reduction in Viral RNA Copy (EC_50_)^d^	90% Reduction in Infectious Virus (EC_90_)^e^
*Repurposed NAs*	Riboprine	>100	1.21 ± 0.04	0.45 ± 0.02	0.48 ± 0.03	1.71 ± 0.05
	Forodesine	>100	1.69 ± 0.06	0.70 ± 0.03	0.73 ± 0.04	2.20 ± 0.06
	Nelarabine	>100	4.14 ± 0.14	1.68 ± 0.08	1.75 ± 0.08	6.46 ± 0.18
	Tecadenoson	>100	7.73 ± 0.23	2.90 ± 0.10	2.95 ± 0.11	11.93 ± 0.32
	Maribavir	>100	7.97 ± 0.27	3.05 ± 0.13	3.20 ± 0.14	12.30 ± 0.33
	Vidarabine	>100	8.10 ± 0.25	3.20 ± 0.13	3.23 ± 0.13	12.60 ± 0.35
*Reference Drugs*	Remdesivir	>100	5.99 ± 0.20	2.07 ± 0.08	2.11 ± 0.09	8.01 ± 0.29
	Molnupiravir	>100	6.28 ± 0.28	2.61 ± 0.10	2.73 ± 0.11	9.10 ± 0.31
*Placebo Solvent*	DMSO	>100	>100	>100	>100	>100

^a^CC_50_ or 50% cytotoxic concentration is the concentration of the tested compound that kills half the cells in an uninfected cell culture. CC_50_ was determined with serially-diluted compounds in Vero E6 cells at 48 h postincubation using CellTiter-Glo Luminescent Cell Viability Assay (Promega)

^b^CPEIC_100_ or 100% CPE inhibitory concentration is the lowest concentration of the tested compound that causes 100% inhibition of the cytopathic effects (CPE) of SARS-CoV-2 B.1.1.529/BA.2 virus in Vero E6 cells under increasing concentrations of the tested compound at 48 h postinfection. Compounds were serially diluted from 100 μM concentration

^c^EC_50_ or 50% effective concentration is the concentration of the tested compound that is required for 50% reduction in infectious SARS-CoV-2 B.1.1.529/BA.2 virus particles in vitro. EC_50_ is determined by infectious virus yield in culture supernatant at 48 h postinfection (log_10_ TCID_50_/mL)

^d^EC_50_ or 50% effective concentration is the concentration of the tested compound that is required for 50% reduction in SARS-CoV-2 B.1.1.529/BA.2 viral RNA copies in vitro. EC_50_ is determined by viral RNA copies number in culture supernatant at 48 h postinfection (log_10_ RNA copies/mL)

^e^EC_90_ or 90% effective concentration is the concentration of the tested compound that is required for 90% reduction in infectious SARS-CoV-2 B.1.1.529/BA.2 virus particles in vitro. EC_90_ is determined by infectious virus yield in culture supernatant at 48 h postinfection (log_10_ TCID_90_/mL)

It was surprisingly observed that riboprine and forodesine successfully act against the SARS-CoV-2 in a relatively rapid mode of action, with their maximal effectiveness against the Omicron variant particles reached within about 4–10 h of starting administration and treatment. Exactly as adenosine and other natural analogs, the triphosphate forms of riboprine and forodesine (riboprine-TP and forodesine-TP), which are pharmacokinetically known to be the major metabolic phosphorylated esters of both drugs, are expected to be as effective as the administered original forms or even much more (due to the higher biocompatibility). Recently, some studies reported similar promising results of the triphosphate metabolites of some NAs but on other subvariants of the SARS-CoV-2 Omicron variant [36]. The current results of this reliable bioassay are in excellent agreement with almost all the findings of the previous anti-RdRp biochemical assay along with the previous computational study (which was discussed in details in Computational molecular modeling of the selected NAs as prospective anti-COVID-19 drugs) of this current comprehensive research.

## Conclusions and future therapeutic recommendations

Recently, nucleoside antivirals topped the scene as first and early choices for COVID-19 therapy [37]. The current comprehensive in silico/in vitro preclinical research study disclosed the anti-COVID-19 potentials of a series of NAs, with riboprine and forodesine being the most promising potent SARS-CoV-2 RNA mutagens or, at least, the most promising coronaviral-2 replication inhibitors in general. Riboprine is a natural purine nucleoside analog (mainly a phytochemical metabolite/plant hormone) investigated for its potential various antineoplastic/antiproliferative, proapoptotic, neuroprotective, and antiangiogenic activities [38], while forodesine is a very potent synthetic and unique highly selective transition-state analog inhibitor of purine nucleoside phosphorylase (PNP), approved and used recently for the effective treatment of relapsed/refractory peripheral T-cell lymphoma [39]. Physically, riboprine and forodesine molecules have very flexible chemical structures that can easily tolerate chemical changes in biological systems. It was clearly found in the current research study that coronaviral-2 particles are very sensitive to both compounds and thoroughly mutated/inhibited by them. Interestingly, it was discovered that riboprine and forodesine may effectively stop SARS-CoV-2 spreadability and pathogenicity (and, consequently, end COVID-19 infection as a whole) in the human body, mainly through severely hindering SARS-CoV-2 replication *via* a double synergistic inhibitory mode of action against the two SARS-CoV-2 enzymes RdRp and ExoN. This double mode of action could be extended to a triple one if the expected inhibitory effects of the two drugs against kinases, specially on ADK, are extensively explored and proved in a next study. Similar to their natural analogs, the triphosphate forms (esters) of riboprine and forodesine are predicted to be as effective as the administered original forms. Based on the current research observations, the two NAs, riboprine and forodesine, are specifically prioritized as prospective COVID-19 therapeutic drugs (with very promising anti-SARS-CoV-2 EC_50_ values of 0.45 and 0.70 μM, respectively, against the Omicron variant), while all the six promising NAs, riboprine, forodesine, nelarabine, tecadenoson, maribavir, and vidarabine, generally warrant deeper pharmacological and clinical investigations to clearly understand their accurate therapeutic values as potential anti-SARS-CoV-2 agents.

## Materials and methods

### In silico computational evaluation

#### Aimed coronaviral-2 proteins preparation

**Fig. 6:**
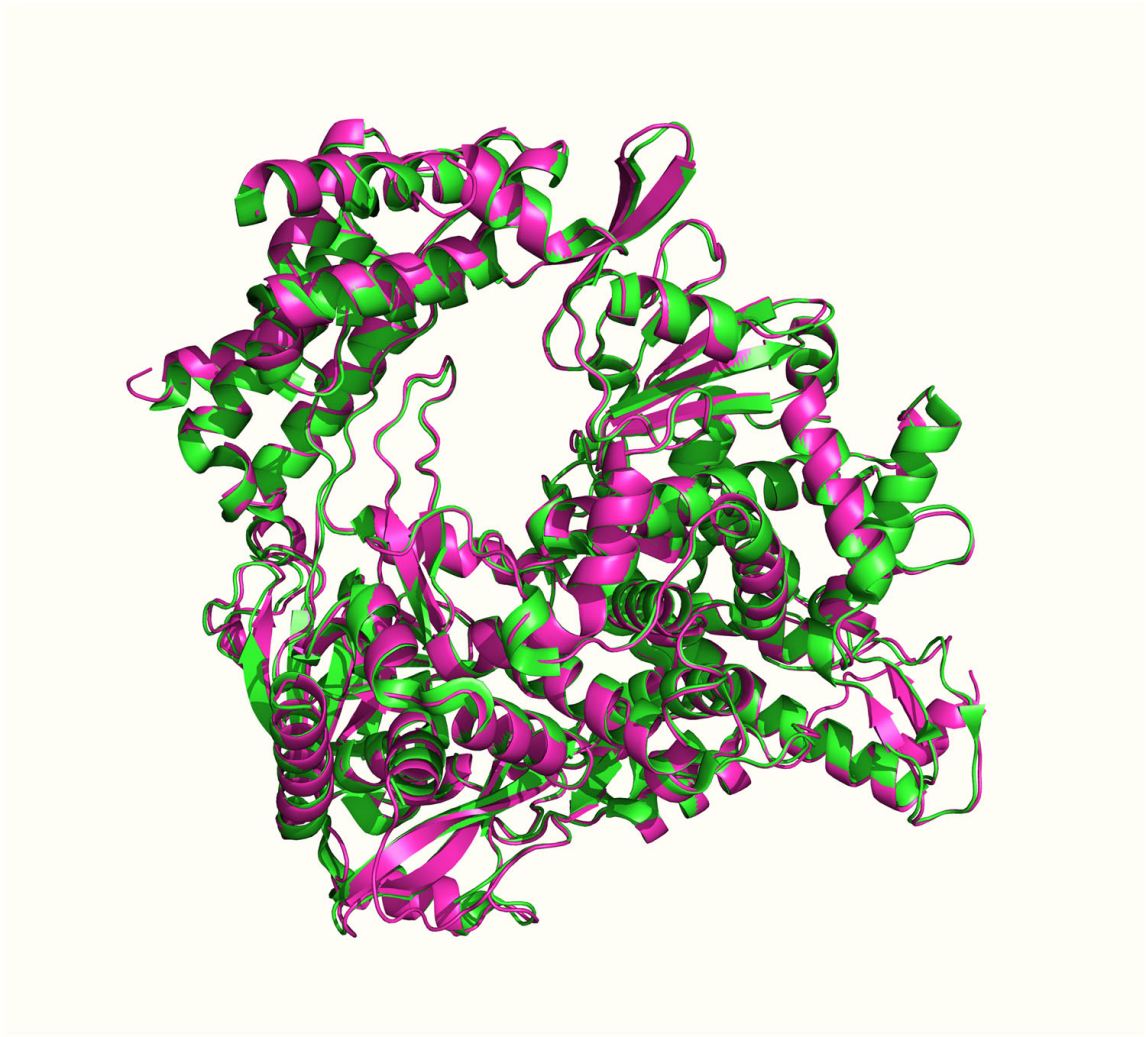
3-D representation of the two PDB files of the SARS-CoV-2 RdRp enzyme, 7OZU (shown in green color) and 7BV2 (shown in magenta color), superposed on each other

The 3-D structures of the target SARS-CoV-2 RdRp and ExoN proteins were obtained from the RCSB PDB with PDB identification (ID) codes 7BV2 (a newer code of SARS-CoV-2 RdRp but cocrystallized with molnupiravir/NHC is now available at the RCSB PDB, the PDB ID code 7OZU. However, we chose to use the PDB ID code 7BV2 due to three main reasons; first, it is the original and most validated SARS-CoV-2 RdRp code, second, the RdRp protein of this code is cocrystallized with the triphosphate form of the principal reference drug of the current study, which is remdesivir, and third, the superposing of the two proteins, 7OZU and 7BV2, shows extremely slight RMSD difference, less than 0.4 Å, which means that the two PDB files are almost identical, as obviously shown in Fig. 6, giving nearly the same results upon molecular docking and MD simulation, and that is originally because 7BV2 was used as a template model when the 7OZU crystal structure has been created) and 7MC6, respectively. Both enzymatic proteins were obtained in the complex forms with their protein cofactors (i.e., were obtained cocrystallized in the nsp12-nsp7-nsp8 and nsp14-nsp10 complex forms, respectively) to increase nature simulation. The PDB files of the two proteins were properly downloaded. Proteins were viewed through Pymol Molecular Graphic Visualizer software 2.4, and their predetected active site residues (with their closest neighboring residues) were then checked for complete presence and correctness. The catalytic active site residues highlighted through Pymol software were noted for the next in silico studies.

#### Target nucleosidic ligands selection and preparation

To choose the best NAs for the current study, a primary virtual screening of diverse libraries of hundreds of NAs was done against SARS-CoV-2 RdRp and ExoN proteins using the Molecular Operating Environment (MOE) platform (Chemical Computing Group). The fifteen NAs with the top collective results as the best hitting candidates of both proteins were selected to continue the long journey of this present research study. After this accurate screening, an extensive literature survey was also performed for the study of the potentials of the chosen fifteen NAs as antivirals. Many of them have demonstrated strong antiviral capabilities either in computational or experimental studies or in both of them. This is one of the main reasons why these potential inhibitors have been experimented in the current virtual docking and simulation studies of SARS-CoV-2 RdRp and ExoN enzymes. Although the standard anti-SARS-CoV-2 NAs, GS-441524 and NHC, gave almost similar results as their pro/parent drugs, remdesivir and molnupiravir, in the primary virtual screening, but we chose to use remdesivir and molnupiravir as the reference drugs for comparison to continue the molecular modeling examinations, this is mainly because, first, both drugs are officially-used and FDA-approved medicines against SARS-CoV-2/COVID-19, not their metabolites, and, second, both drugs would be employed as the reference/control drugs in the assays of the biological evaluations later. The chemical structures of the selected NAs, including the references, were adequately prepared using ChemDraw Professional 16.0 software (licensed version) for the next in silico studies.

#### Molecular docking protocol

Blind docking of the fifteen selected NAs in SARS-CoV-2 RdRp and ExoN proteins was performed *via* MOE. Remdesivir (with its phosphate group in the free form in order to match this major reference drug with the tested NAs as much as possible) and molnupiravir were used as positive control anti-SARS-CoV-2 references having proven potent RdRp/ExoN inhibitory activities. Prior to starting these docking procedures, some important preparations (mainly, additions and corrections) were required. All the missed atoms/residues in the SARS-CoV-2 RdRp and ExoN structures were added *via* MOE structure modeling. The two specific proteins were further precisely prepared for molecular docking by the addition of hydrogen atoms using the 3D-protonation module of the used MOE software; any partial charges were also corrected for both proteins. RdRp and ExoN proteins were energy minimized in their complex forms *via* the Amber-99 force field which is available in MOE. Similarly, the structures of the fifteen target ligands, remdesivir, and molnupiravir were also adequately energy minimized in MOE. For docking of the target/reference ligands with the two proteins, the known London-dG scoring functions were utilized for binding energy calculations. For each docked NA/reference molecule, the MOE software produced about twenty different poses with each docked SARS-CoV-2 protein. Of all the docking poses for each molecule with each protein, the one with the highest number of best molecular interactions, i.e., the top ranked pose of the best interactions, was recorded and saved. MOE gives a numerical value for the interaction of any potential ligand with any certain protein in the form of docking S-score (docking scores are expressed in kcal/mol). This docking binding energy or S-score represents the net energy of the formed protein-ligand complex and it also primarily reflects the degree of its expected stability (i.e., it provides a primary idea about the predicted stability of this formed complex prior to performing the more detailed robust computations *via* the MD simulations). The molecular docking revealed six promising target NAs with very good S-scores compared to the two reference NAs (these top ranked NAs represent the core point of the current research). MOE software shows all the possible molecular interactions (of all types) made during the docking process; these include, e.g., H-bonds, hydrophobic interactions, ionic interactions/bonds, and salt bridges. For the best six target NAs and the two reference NAs, the 2-D and 3-D output files/images of all the produced protein-ligand complexes (showing almost all the possible interactions) were saved for reporting and further investigative analysis.

#### MD simulation protocol

The six NAs ranked with the top results, e.g., with the best molecular interactions, lowest docking score (S-score), and lowest RMSD, computed through MOE (using the apoenzymes of RdRp and ExoN for comparison purposes) against both proteins were then employed for further in silico studies, mainly the MD simulation studies, using Schrodinger’s Desmond module MD-Simulation software. For MD simulation of the selected NAs, the best docking poses of these NAs in complexes with the SARS-CoV-2 RdRp and ExoN enzymes were kept in PDB format in MOE to be used for further virtual stability studies in Schrodinger’s Desmond module. The in-built Desmond System Builder tool was used in this current protocol to create the solvated water-soaked MD-Simulation system. The TIP3P model was utilized as the solvating model in the present experiment. With periodic boundary conditions, an orthorhombic box was accurately simulated with a good boundary distance of at least 10 Å from the outer surface of each of the two coronaviral-2 proteins. The simulation systems were neutralized of complex charges by the addition of a reasonably sufficient amount of counter ions. The isosmotic state was maintained by adding 0.10 mol/L sodium and chloride ions, i.e., 0.10 M NaCl, into the simulation panel to keep imitating the actual isosmotic conditions during all simulations. Prior to beginning the simulation process, a predefined equilibration procedure was done. The system of the MD simulation was equilibrated by employing the standard Desmond protocol at a constant pressure of 1.0 bar and a constant temperature of 300 K (NPT ensemble; considering the viral nature of the two target enzymatic proteins), and also by employing the known Berendsen coupling protocol with one temperature group. Hydrogen atom bond length was properly constrained using the validated SHAKE algorithm. Particle Mesh Ewald (PME) summation method was used to specifically model long-range electrostatic interactions. On the other hand, an exact cutoff of 10 Å was specifically assigned for van der Waals and short-range electrostatic interactions. As previously mentioned, the MD simulation was run at ambient pressure conditions of about 1.013 bar while the used temperature was exactly set to 300 K for each 100 nsec (ns) period of this MD simulation, and 1000 frames were saved into the simulation trajectory file. The simulation run time for each complex system and apo system was fixed to 100 ns as a total. After simulations, the trajectory files of the simulated systems were used for calculation of the various structural parameters required, e.g., RMSD (Å), RMSF (Å), rGyr (Å), number of protein-ligand contacts (# of total contacts), interactions fractions (%), intermolecular H-bonds (from all aspects), MolSA (Å^2^), SASA (Å^2^), and PSA (Å^2^), to extensively perform stability studies of the complex and apo systems. The results of the most promising two compounds, riboprine and forodesine, were saved to be reported and discussed in the current paper.

### In vitro biological evaluation

#### Specifications of the bioexamined NAs

Riboprine (*N*^6^-(2-Isopentenyl)adenosine, CAS Registry Number: 7724-76-7) was purchased from BenchChem (BENCH CHEMICAL, Austin, Texas, U.S.A.) (Catalog Number: B141774, Purity: ≥ 99%). While forodesine (Immucillin-H, CAS Registry Number: 209799-67-7), nelarabine (Arranon, CAS Registry Number: 121032-29-9), tecadenoson (CVT-510, CAS Registry Number: 204512-90-3), maribavir (1263W94, CAS Registry Number: 176161-24-3), vidarabine (Arabinosyladenine “Ara-A”, CAS Registry Number: 5536-17-4), remdesivir (GS-5734, CAS Registry Number: 1809249-37-3), and molnupiravir (EIDD-2801, CAS Registry Number: 2349386-89-4) were purchased from Biosynth Carbosynth (Carbosynth Ltd., Berkshire, U.K.) (for forodesine, Product Code: MD11591, Purity: ≥ 98%; for nelarabine, Product Code: NN26176, Purity: ≥ 98%; for tecadenoson, Product Code: EIA51290, Purity: ≥ 98%; for maribavir, Product Code: AM178224, Purity: ≥ 98%; for vidarabine, Product Code: NA06007, Purity: ≥ 98%; for remdesivir, Product Code: AG170167, Purity: ≥ 98%; for molnupiravir, Product Code: AE176721, Purity: ≥ 98%). The ultrapure solvent DMSO (CAS Registry Number: 67-68-5) was purchased from a local distributor, El-Gomhouria Company For Drugs (El-Gomhouria Co. For Trading Drugs, Chemicals & Medical Supplies, Mansoura Branch, Egypt) (Purity: ≥ 99.9% “anhydrous”).

#### In vitro anti-RdRp/anti-ExoN assay (SARS-CoV-2-RdRp-Gluc reporter assay) of the selected NAs

First, the used cells, 293T cells (ATCC CRL-3216), were kept in Dulbecco’s modified Eagle’s medium (DMEM; Gibco) with 10% (v/v) fetal bovine serum (FBS; Gibco), then they were cultured at 37 °C in a humidified atmosphere of CO_2_ (5%). HEK293T cells were transfected using Vigofect transfection reagents (Vigorous) according to the strict instructions of the manufacturer. The required plasmid DNAs, antibodies, and reagents were purchased and treated exactly as in the literature procedures [24, 25]. The tested drugs are as described and specified in Specifications of the bioexamined NAs. Also, western blotting (for the collected transfected HEK293T cells), real-time RT-PCR (for the extracted total RNA of transfected HEK293T cells), and cell viability test (using Cell Counting Kit-8 (CCK8), Beyotime) were exactly performed as the typical procedures of the literature [24, 25]. The steps of the well-designed in vitro SARS-CoV-2-RdRp-Gluc reporter assay were accurately carried out according to the same original method of literature but with modifying almost all the proteins to be pertinent and identical to the SARS-CoV-2 Omicron variant “B.1.1.529/BA.2 sublineage” (HEK293T cells were transfected in this biochemical assay with CoV-Gluc, nsp12, nsp7, and nsp8 plasmid DNAs at the ratio of 1:10:30:30, and with CoV-Gluc, nsp12, nsp7, nsp8, nsp10, and nsp14 plasmid DNAs at the ratio of 1:10:30:30:10:90) [24, 25]. Exactly as instructed in the original assay, a stock of coelenterazine-h was dissolved in absolute ethanol (of very pure analytical grade) to a concentration of 1.022 mM [24, 25]. Directly before each assay, the stock was diluted in phosphate-buffered saline (PBS) to a concentration of 16.7 μM and incubated in the dark for 30 min at room temperature [24, 25]. For luminescence assay, 10 μL of supernatant was added to each well of a white and opaque 96-well plate, then 60 μL of 16.7 μM coelenterazine-h was injected, and luminescence was measured for 0.5 s using the Berthold Centro XS3 LB 960 microplate luminometer [24, 25]. Final results were statistically represented as the mean (*µ*) ± the standard deviation (*SD*) from at least three independent experiments. Statistical analysis was performed using SkanIt 4.0 Research Edition software (Thermo Fisher Scientific) and Prism V5 software (GraphPad). All resultant data were considered statistically significant at *p* < 0.05.

#### In vitro anti-SARS-CoV-2 and cytotoxic bioactivities multiassay of the selected NAs

This validated in vitro anti-COVID-19 multiassay (including the cytotoxicity test), which was designed for the assessment of the net anti-SARS-CoV-2 activities of potential anti-COVID-19 agents, is based mainly upon the authentic procedures of Rabie [5, 13, 14, 16–19]. The complete procedures were carried out in a specialized biosafety level 3 (BSL-3) laboratory. The assayed new strain of SARS-CoV-2 virus, the Omicron variant, B.1.1.529/BA.2 sublineage, was isolated from the fresh nasopharynx aspirate and throat swab of a 43.5-year-old Indian man with confirmed COVID-19 infection using Vero E6 cells (ATCC CRL-1586) on 4 February, 2022. The starting titer of the stock virus (10^7.25^ TCID_50_/mL) was prepared after three serial passages in Vero E6 cells in infection media (DMEM supplemented with 4.5 g/L D-glucose, 100 mg/L sodium pyruvate, 2% FBS, 100 000 U/L Penicillin-Streptomycin, and 25 mM *N*-(2-hydroxyethyl)piperazine-*N*′-ethanesulfonic acid (HEPES)). The tested target and reference NAs are as described and specified in Specifications of the bioexamined NAs. Preliminary pilot assays were performed mainly to determine the best concentration of the tested NAs to begin the in vitro anti-SARS-CoV-2 and cytotoxicity tests with. Accordingly, the stocks of the tested compounds were precisely prepared by dissolving each of the eight compounds in DMSO to obtain a 100 μM concentration of each compound. Additionally, DMSO was used for the purpose of a negative control comparison to make this experimental study placebo-controlled. To assess the total in vitro anti-SARS-CoV-2 activity of each of the target drugs, riboprine, forodesine, nelarabine, tecadenoson, maribavir, and vidarabine, in comparison to that of each of the two positive control/reference drugs, remdesivir and molnupiravir, along with that of the negative control solvent, DMSO, Vero E6 cells were pretreated with each of the nine compounds diluted in infection media for 1 h prior to infection by the new Omicron variant of the SARS-CoV-2 virus at MOI = 0.02. The nine tested compounds were maintained with the virus inoculum during the 2 h incubation period. The inoculum was removed after incubation, and the cells were overlaid with infection media containing the diluted test compounds. After 48 h of incubation at 37 °C, supernatants were immediately collected to quantify viral loads by TCID_50_ assay or quantitative real-time RT-PCR “qRT-PCR” (TaqMan Fast Virus 1-Step Master Mix). Viral loads in this assay were fitted in logarithm scale (log_10_ TCID_50_/mL, log_10_ TCID_90_/mL, and log_10_ viral RNA copies/mL), not in linear scale, under increasing concentrations of the tested compounds. Four-parameter logistic (4PL) regression (GraphPad Prism) was used to fit the dose-response curves and determine the EC_50_ and EC_90_ of the tested compounds that inhibit SARS-CoV-2 viral replication (CPEIC_100_ was also determined for each compound). Cytotoxicity of each of the nine tested compounds was also evaluated in Vero E6 cells using the CellTiter-Glo Luminescent Cell Viability Assay (Promega). Final results were statistically represented as the *µ* ± *SD* from at least three independent experiments. Statistical analysis was done using SkanIt 4.0 Research Edition software (Thermo Fisher Scientific) and Prism V5 software (GraphPad). All produced data were considered statistically significant at *p* < 0.05.

## Supplementary Information

Supplementary Material
