# Supplementary material for: Evaluation of a series of nucleoside analogs as effective anticoronaviral-2 drugs against the Omicron-B.1.1.529/BA.2 subvariant: A repurposing research study
Source: Med Chem Res. 2022 Dec 29;32(2):326–41. doi: 10.1007/s00044-022-02970-3 (PMC9797896; doi:10.1007/s00044-022-02970-3)
Supplement: Supplementary file 1 — Supplementary Material [file 44_2022_2970_MOESM1_ESM.docx]

# *Supplementary Material*

# Evaluation of a series of nucleoside analogs as effective anticoronaviral-2 drugs against the Omicron-B.1.1.529/BA.2 subvariant: A repurposing research study

***Authors' Names & Affiliations*:**

**Amgad M. Rabie^a,b,*^ and Mohnad Abdalla^c,**^**

**^a^ Dr. Amgad Rabie's Research Lab. for Drug Discovery (DARLD), Mansoura City 35511, Mansoura, Dakahlia Governorate, Egypt**

**^b^ Head of Drug Discovery & Clinical Research Department, Dikernis General Hospital (DGH), Magliss El-Madina Street, Dikernis City 35744, Dikernis, Dakahlia Governorate, Egypt**

**^c^ Key Laboratory of Chemical Biology (Ministry of Education), Department of Pharmaceutics, School of Pharmaceutical Sciences, Cheeloo College of Medicine, Shandong University, 44 Cultural West Road, Shandong Province, 250012, PR China**

*** *Principal Corresponding Author*:**

**Dr. Amgad M. Rabie**

**E-mails:** [**amgadpharmacist1@yahoo.com**](mailto:amgadpharmacist1@yahoo.com)**,** [**dr.amgadrabie@gmail.com**](mailto:dr.amgadrabie@gmail.com)

**ORCID iD: 0000-0003-3681-114X**

**Postal Address: Dr. Amgad M. Rabie, 16 Magliss El-Madina Street, Dikernis City 35744, Dikernis, Dakahlia Governorate, Egypt**

**Mobile No.: 002-01019733188 & 002-01112900494 (Egypt)**

**** *Second Corresponding Author*:**

**Dr. Mohnad Abdalla**

**E-mails:** [**mohnadabdalla200@gmail.com**](mailto:mohnadabdalla200@gmail.com)

**ORCID iD: 0000-0002-1682-5547**


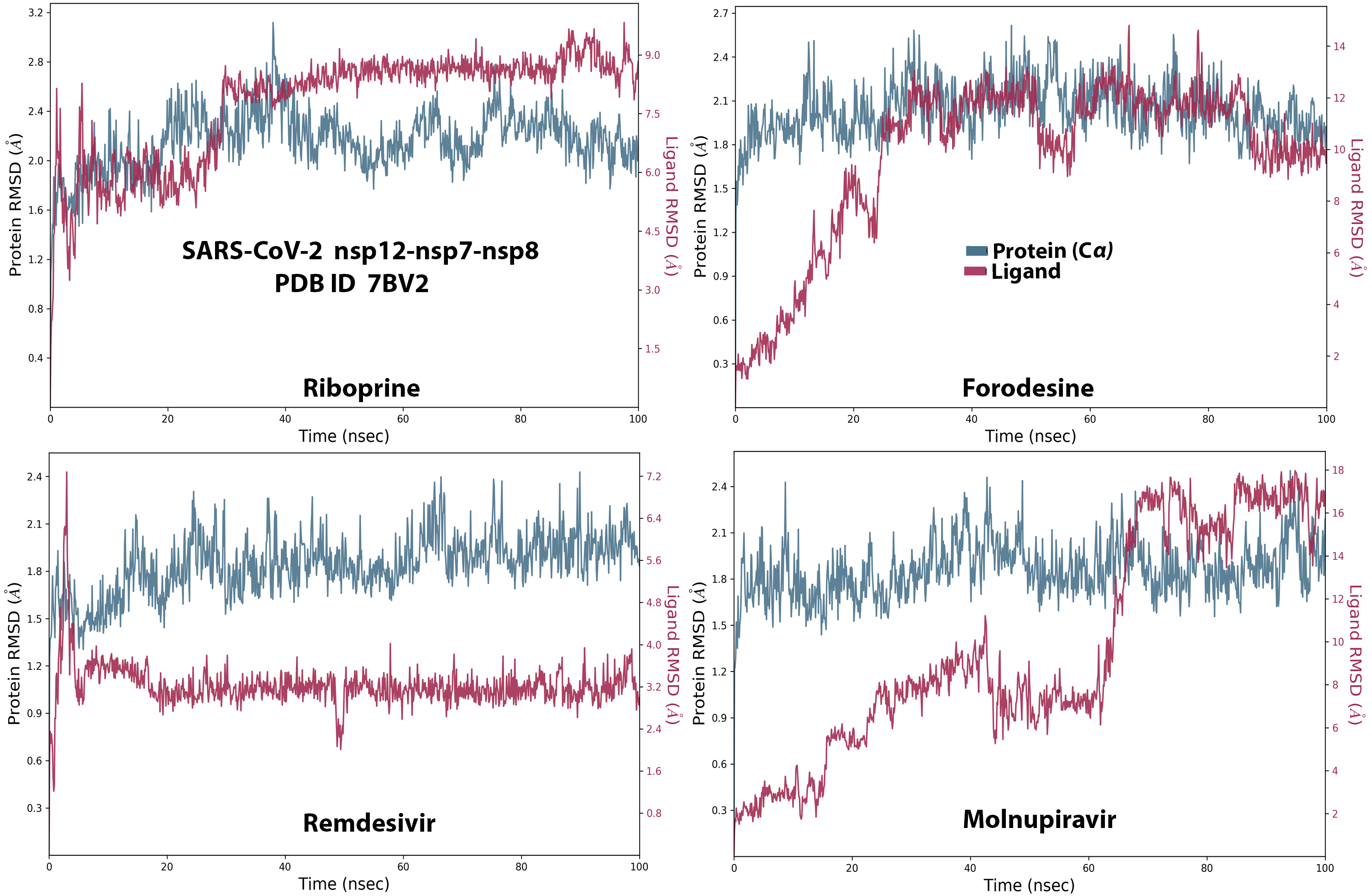


**Fig. S1.** RMSD trajectories (during a simulation period of 100 ns) of the *α*-carbon of amino acid residues of the protein (blue color) and the ligand (maroon color) in the protein-ligand complexes of the two NAs, riboprine and forodesine, and the two reference drugs, remdesivir and molnupiravir, respectively, with the SARS-CoV-2 RdRp "nsp12" enzyme cocrystallized with its protein cofactors nsp7 and nsp8 (PDB ID: 7BV2).


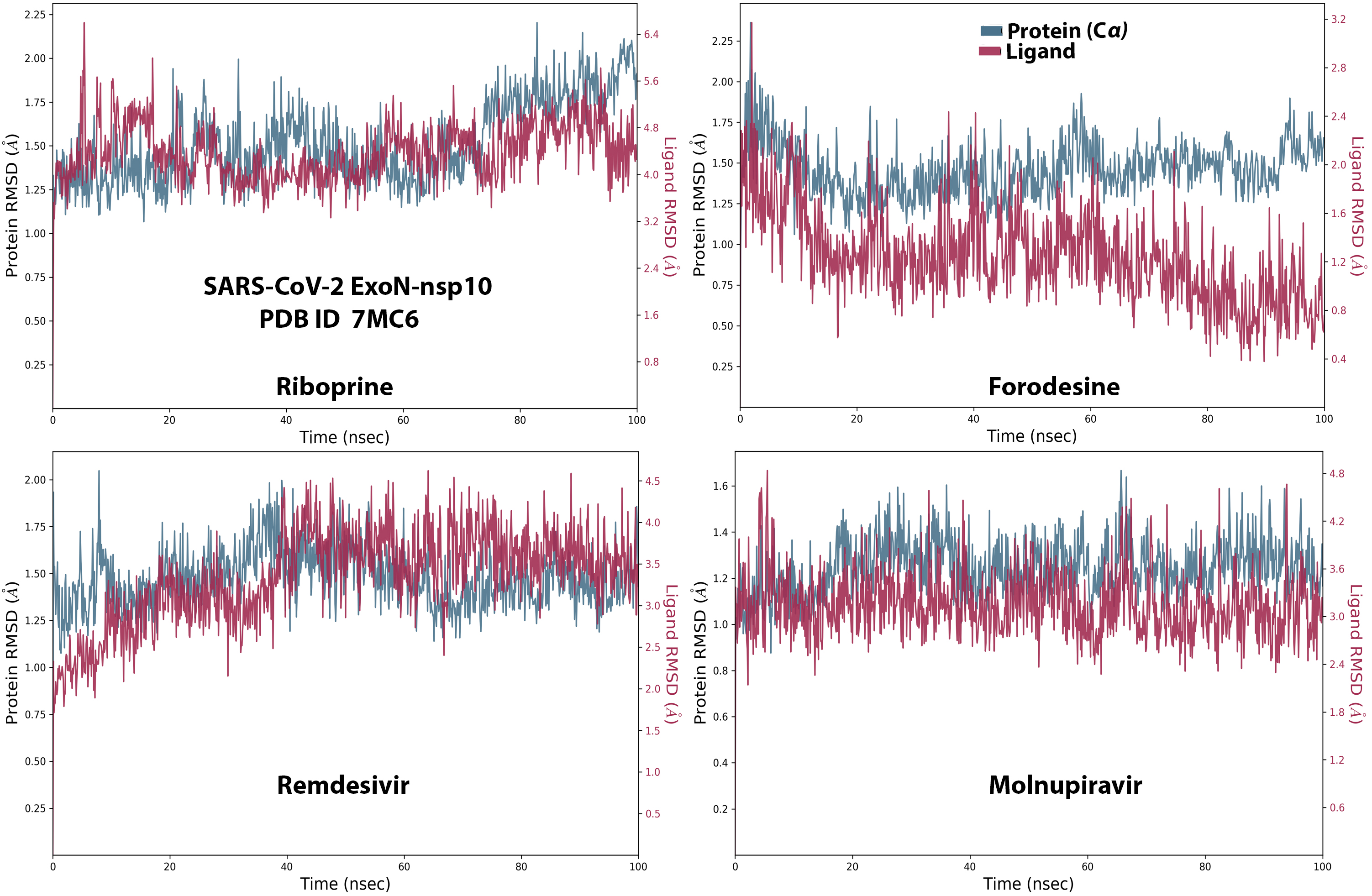


**Fig. S2.** RMSD trajectories (during a simulation period of 100 ns) of the *α*-carbon of amino acid residues of the protein (blue color) and the ligand (maroon color) in the protein-ligand complexes of the two NAs, riboprine and forodesine, and the two reference drugs, remdesivir and molnupiravir, respectively, with the SARS-CoV-2 ExoN "nsp14" enzyme cocrystallized with its protein cofactor nsp10 (PDB ID: 7MC6).


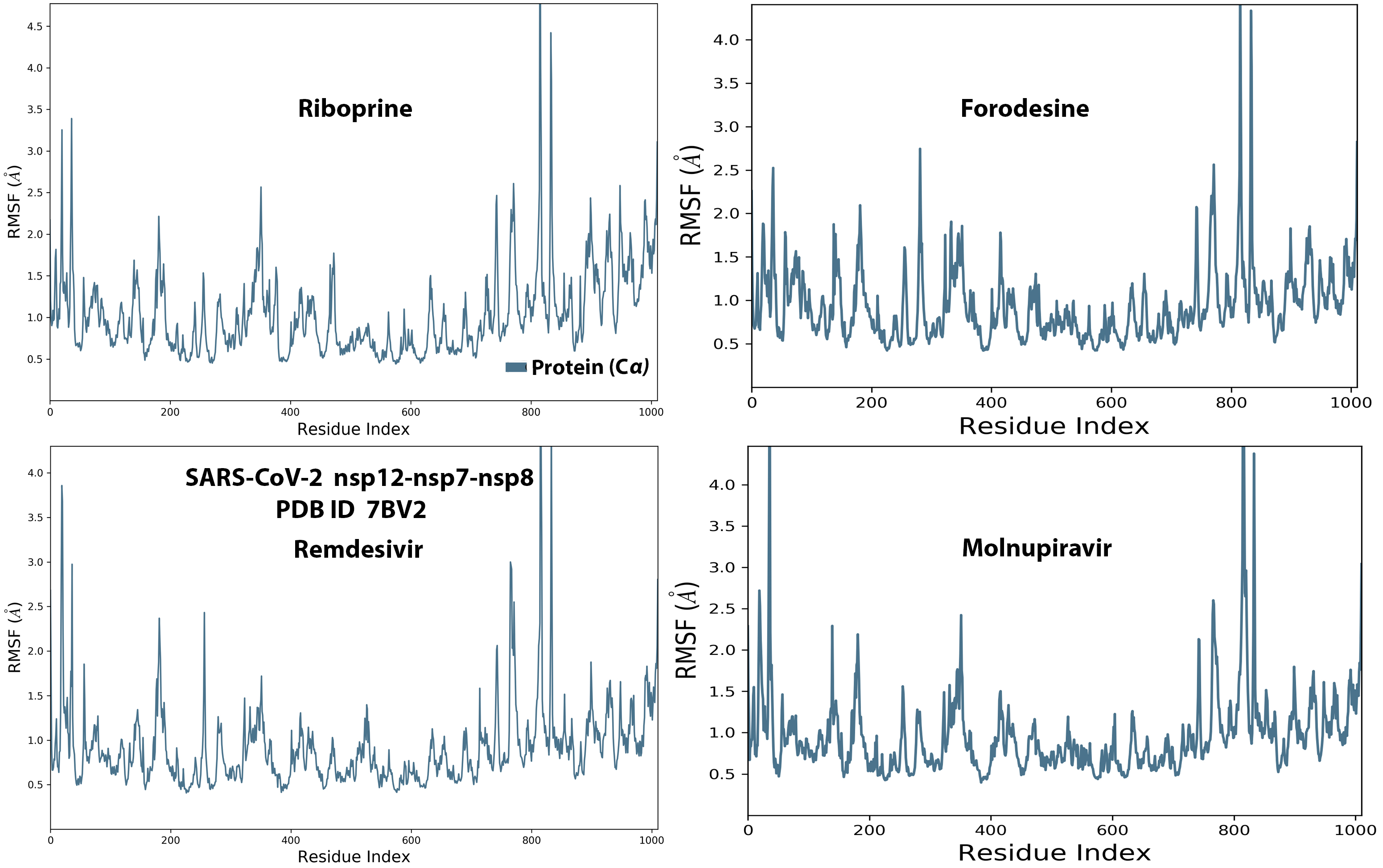


**Fig. S3.** RMSF trajectories (along the different residue regions) of the *α*-carbon of amino acid residues of the protein in the protein-ligand complexes of the two NAs, riboprine and forodesine, and the two reference drugs, remdesivir and molnupiravir, respectively, with the SARS-CoV-2 RdRp "nsp12" enzyme cocrystallized with its protein cofactors nsp7 and nsp8 (PDB ID: 7BV2).


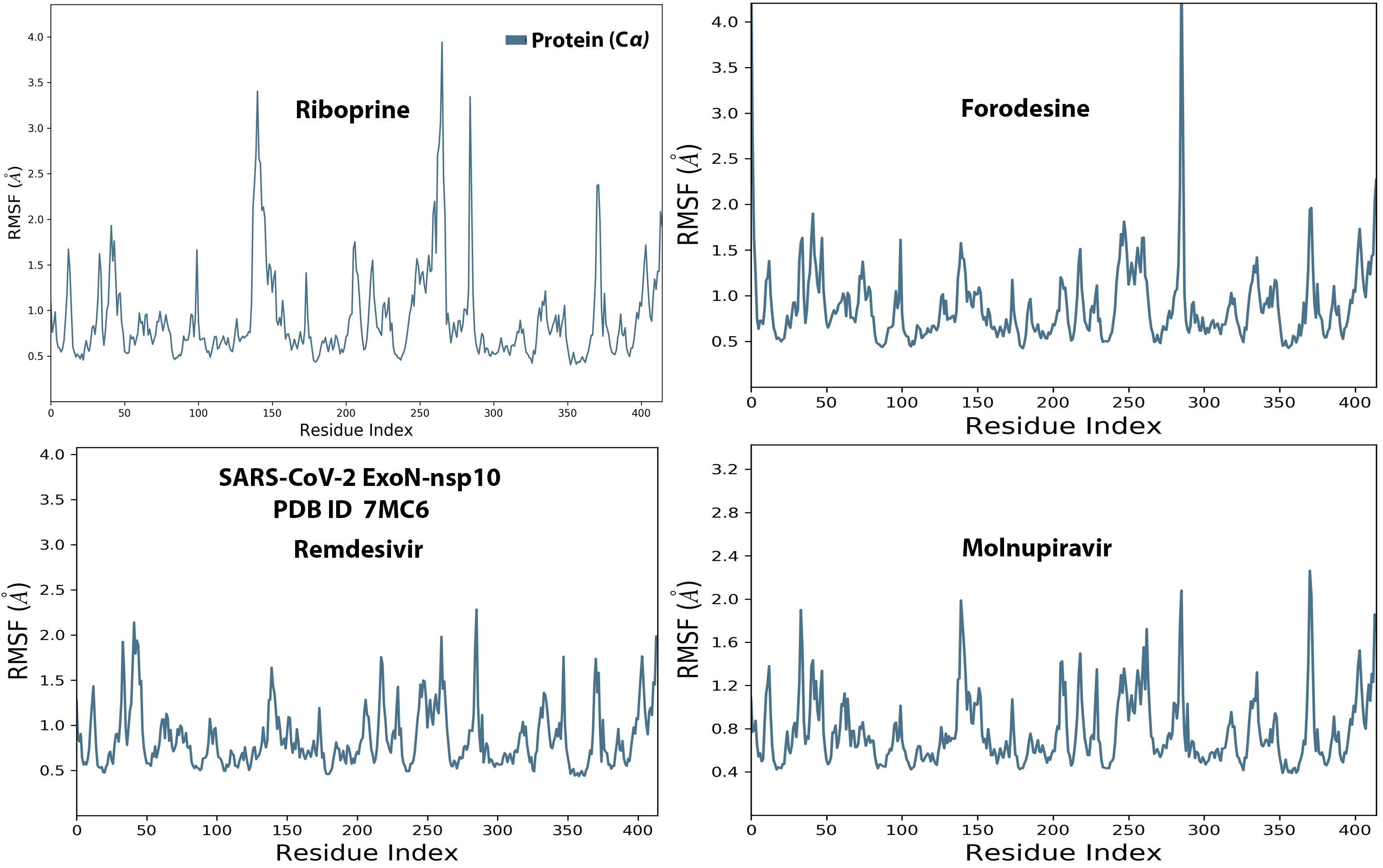


**Fig. S4.** RMSF trajectories (along the different residue regions) of the *α*-carbon of amino acid residues of the protein in the protein-ligand complexes of the two NAs, riboprine and forodesine, and the two reference drugs, remdesivir and molnupiravir, respectively, with the SARS-CoV-2 ExoN "nsp14" enzyme cocrystallized with its protein cofactor nsp10 (PDB ID: 7MC6).


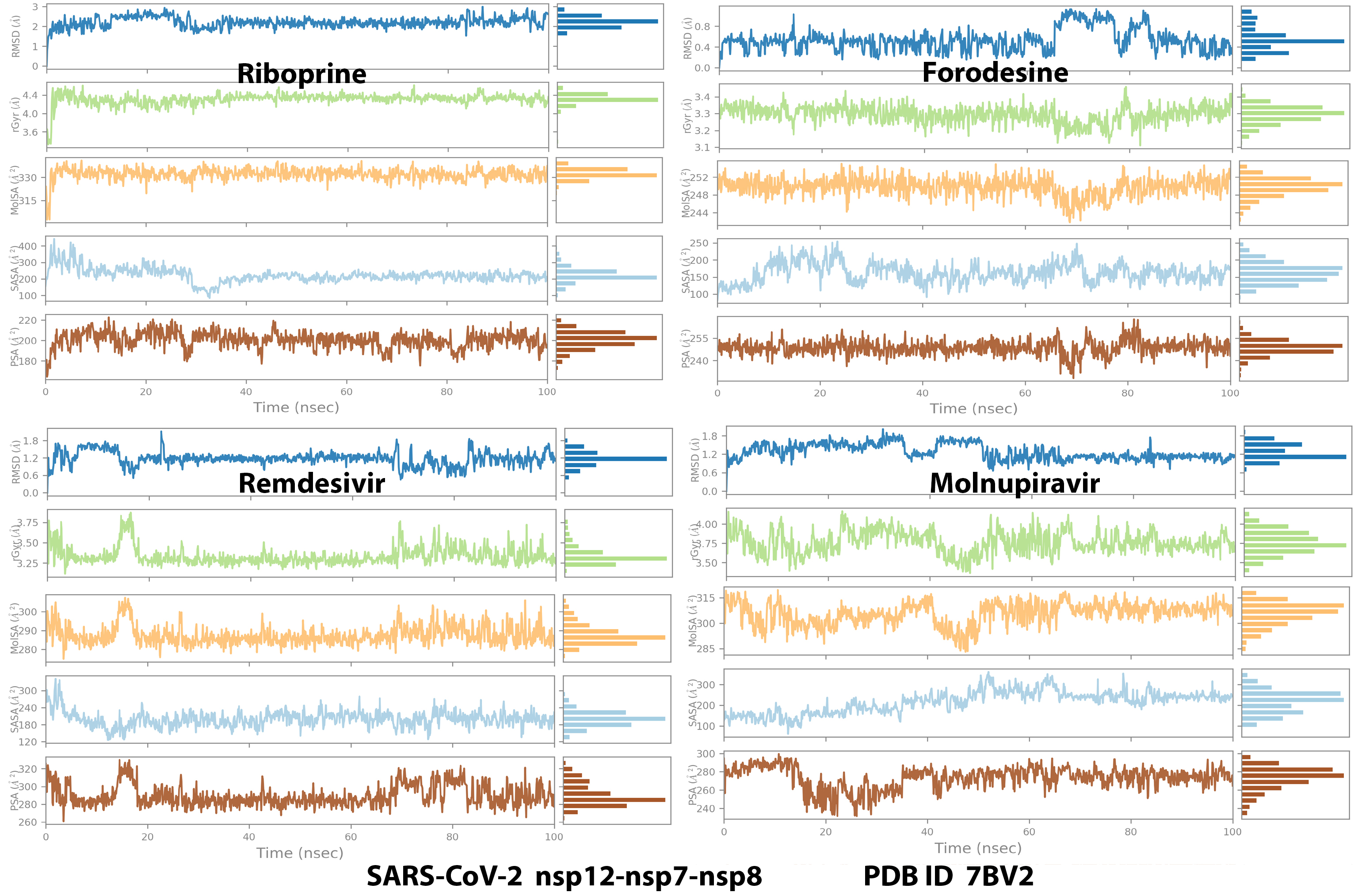


**Fig. S5.** Collective post-MD simulation analysis of the protein-ligand complexes properties (RMSD, rGyr, MolSA, SASA, and PSA) of the two NAs, riboprine and forodesine, and the two reference drugs, remdesivir and molnupiravir, respectively, with the SARS-CoV-2 RdRp "nsp12" enzyme cocrystallized with its protein cofactors nsp7 and nsp8 (PDB ID: 7BV2).


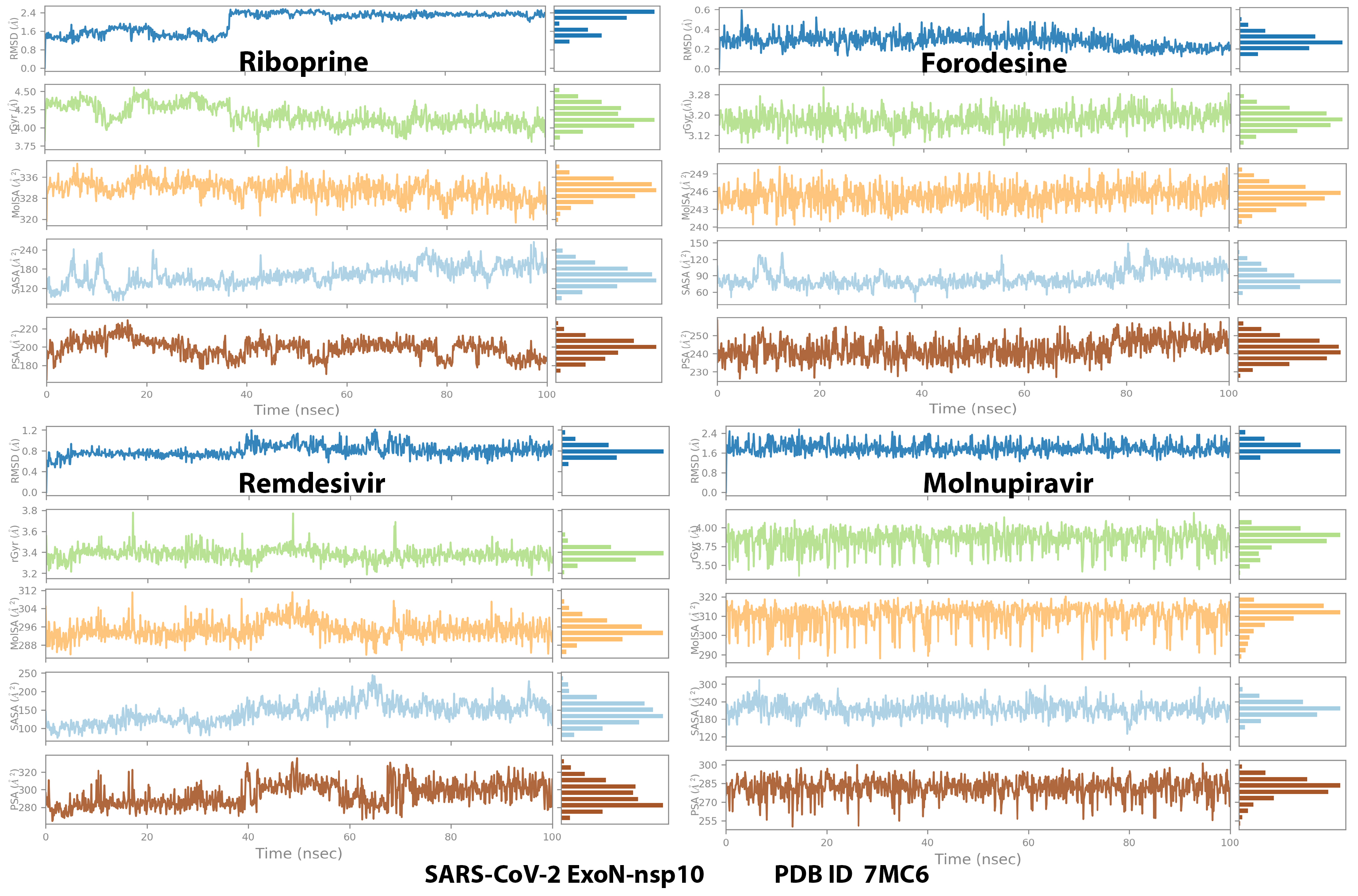


**Fig. S6.** Collective post-MD simulation analysis of the protein-ligand complexes properties (RMSD, rGyr, MolSA, SASA, and PSA) of the two NAs, riboprine and forodesine, and the two reference drugs, remdesivir and molnupiravir, respectively, with the SARS-CoV-2 ExoN "nsp14" enzyme cocrystallized with its protein cofactor nsp10 (PDB ID: 7MC6).


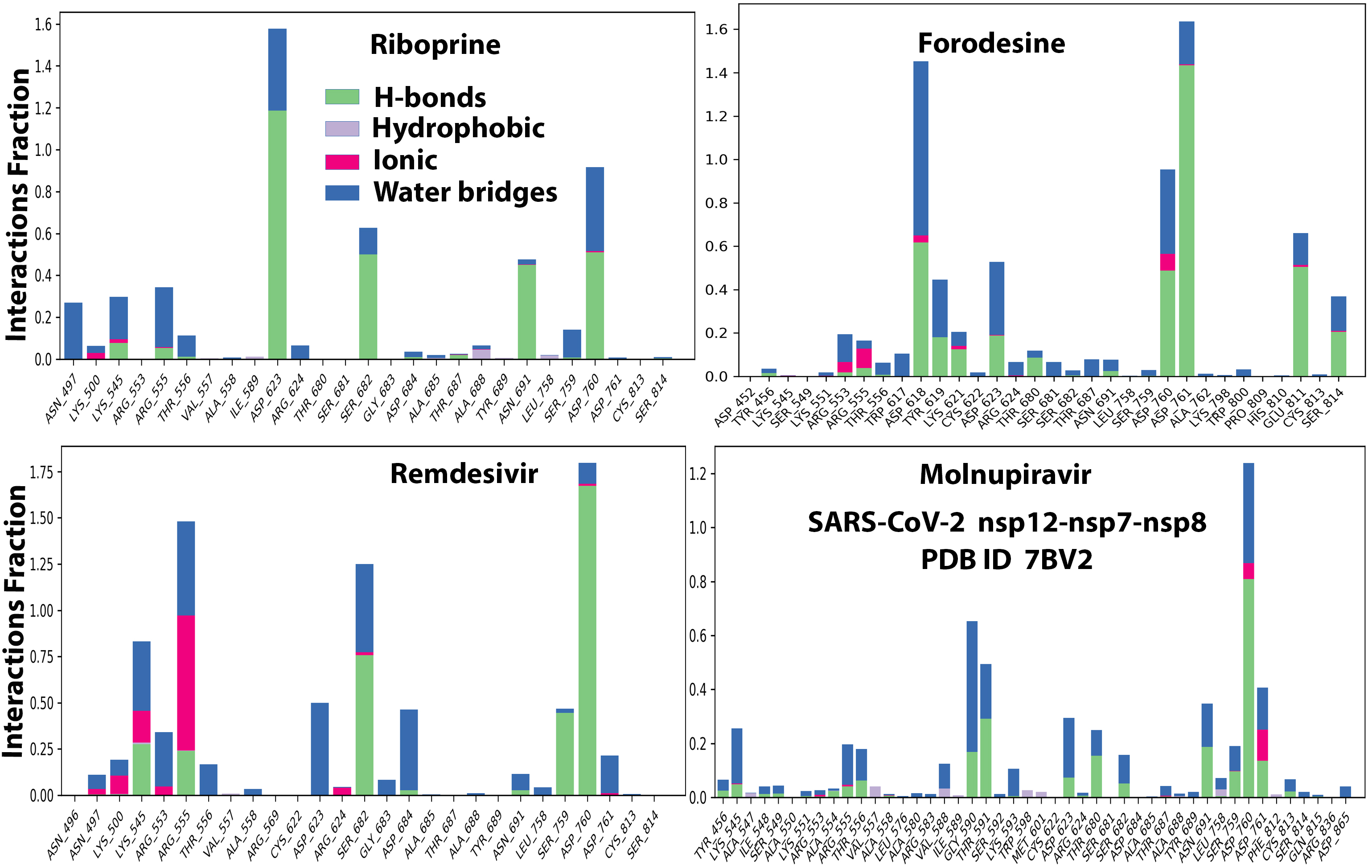


**Fig. S7.** Histograms of the protein-ligand interactions fractions throughout the simulative interaction trajectories of the two NAs, riboprine and forodesine, and the two reference drugs, remdesivir and molnupiravir, respectively, with the SARS-CoV-2 RdRp "nsp12" enzyme cocrystallized with its protein cofactors nsp7 and nsp8 (PDB ID: 7BV2).


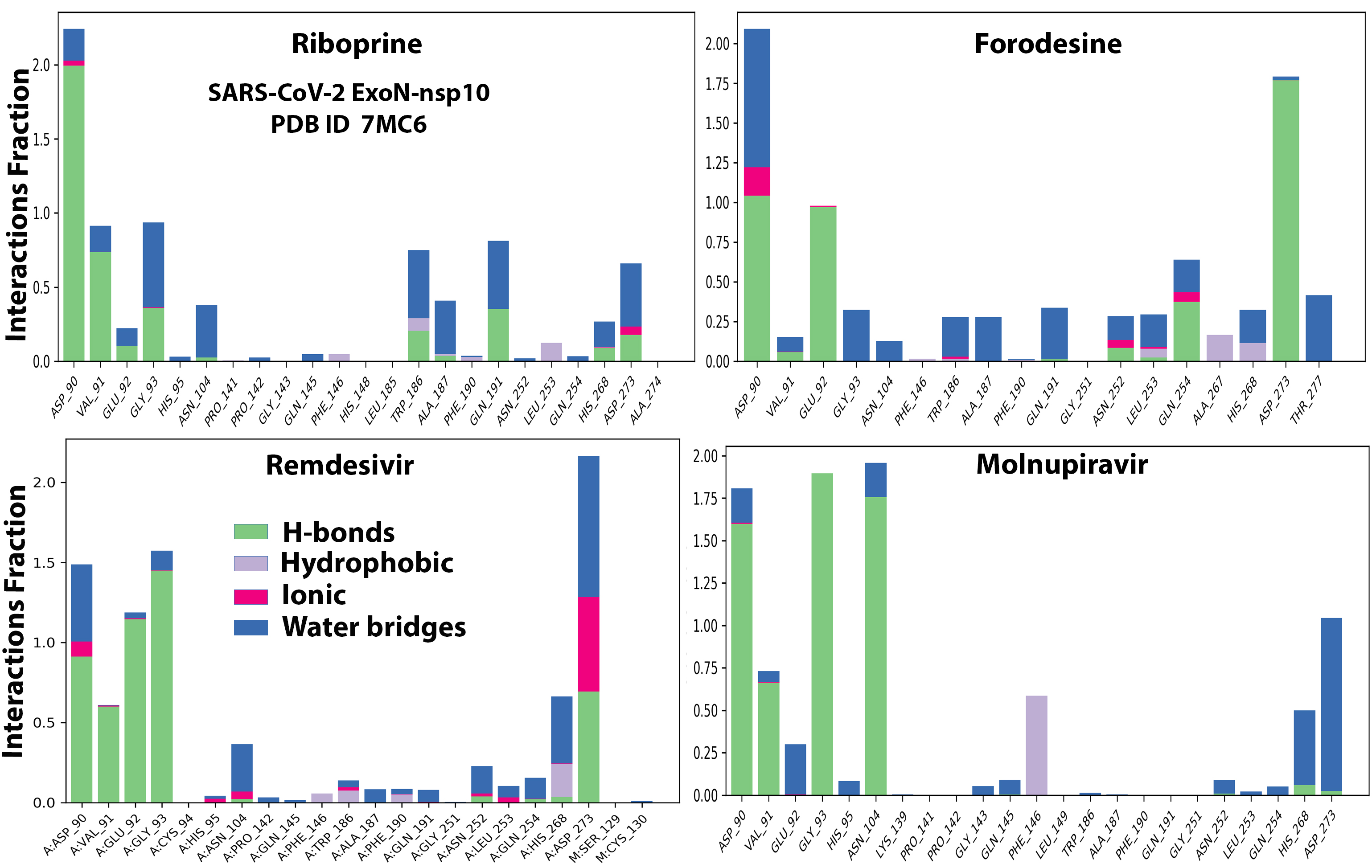


**Fig. S8.** Histograms of the protein-ligand interactions fractions throughout the simulative interaction trajectories of the two NAs, riboprine and forodesine, and the two reference drugs, remdesivir and molnupiravir, respectively, with the SARS-CoV-2 ExoN "nsp14" enzyme cocrystallized with its protein cofactor nsp10 (PDB ID: 7MC6).


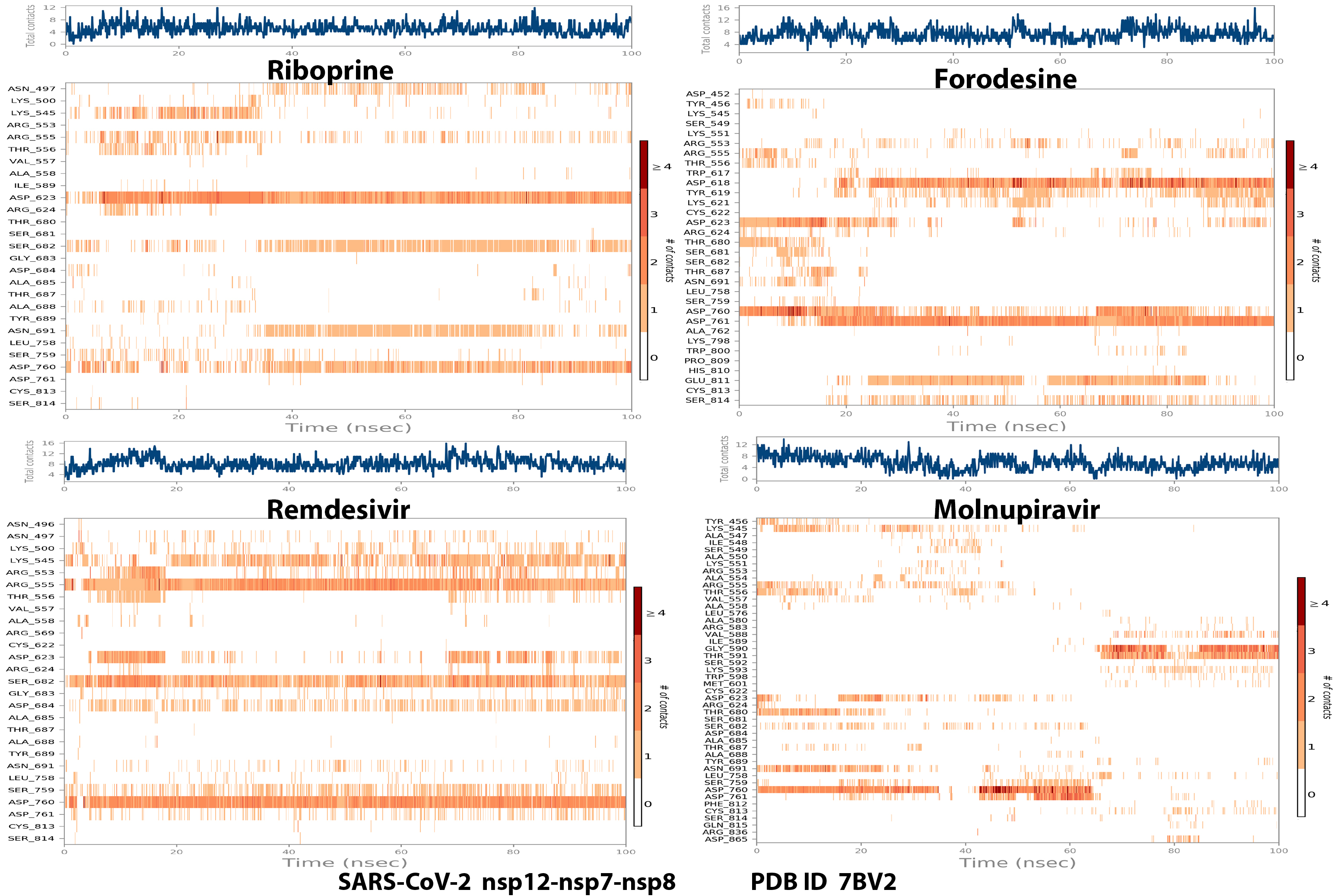


**Fig. S9.** Plots of the distribution of the total number of interactions (contacts) in each trajectory framework of the protein-ligand complexes of the two NAs, riboprine and forodesine, and the two reference drugs, remdesivir and molnupiravir, respectively, with the SARS-CoV-2 RdRp "nsp12" enzyme cocrystallized with its protein cofactors nsp7 and nsp8 (PDB ID: 7BV2).


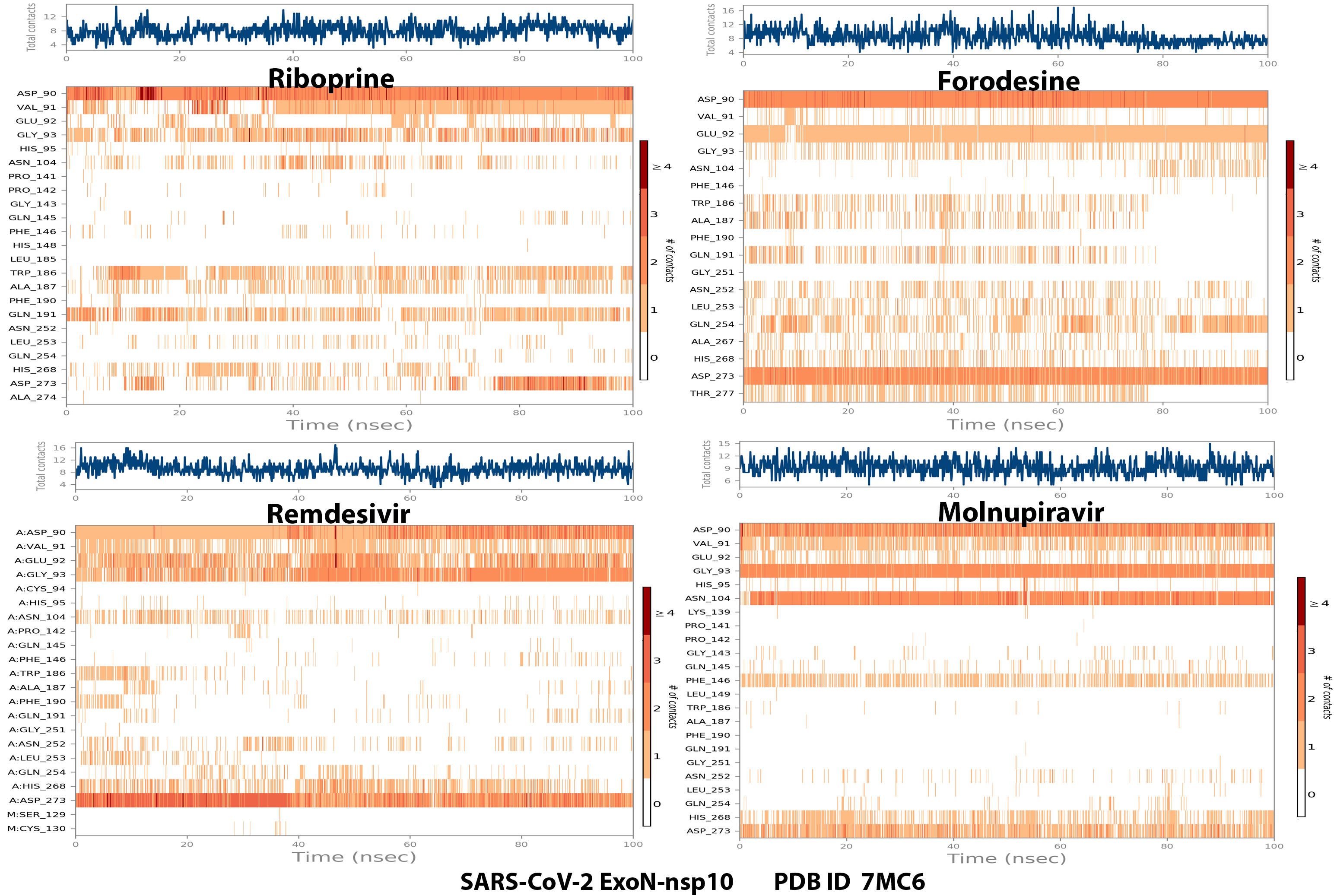


**Fig. S10.** Plots of the distribution of the total number of interactions (contacts) in each trajectory framework of the protein-ligand complexes of the two NAs, riboprine and forodesine, and the two reference drugs, remdesivir and molnupiravir, respectively, with the SARS-CoV-2 ExoN "nsp14" enzyme cocrystallized with its protein cofactor nsp10 (PDB ID: 7MC6).
